# Tricuspid valve repair concomitant with mitral valve surgery: a systematic review and meta-analysis

**DOI:** 10.1097/JS9.0000000000000396

**Published:** 2023-06-07

**Authors:** Kang Yi, Wei Wang, Jianguo Xu, Xin Zhang, Wenxin Wang, Chengfei Liu, Xinyao Li, Tao You

**Affiliations:** a Department of Cardiovascular Surgery, Gansu Provincial Hospital; b Gansu International Scientific and Technological Cooperation Base of Diagnosis and Treatment of Congenital Heart Disease, Lanzhou, Gansu Province; c Department of Cardiac Surgery, The First Hospital of China Medical University, Shenyang, Liaoning; d Evidence-Based Medicine Center, School of Basic Medical Sciences, Lanzhou University; e The First School of Clinical Medicine of Gansu University of Chinese Medicine; f The First Clinical Medical College of Lanzhou University, Lanzhou, China

**Keywords:** meta-analysis, mitral valve surgery, tricuspid regurgitation, tricuspid valve repair

## Abstract

Uncertainties persist about whether to aggressively and effectively treat tricuspid regurgitation (TR) during mitral valve (MV) surgery.

**Review methods:**

Systematic literature searches were performed in five databases to collect all relevant studies published before May 2022 on whether the tricuspid valve was treated during MV surgery. Separate meta-analyses were performed on data from unmatched studies and randomized controlled trials (RCT)/adjusted studies.

**Main results:**

A total of 44 publications were included, of which eight were RCT studies and the rest were retrospective studies. There was no difference in 30-day mortality [odds ratio (OR): 1.00, 95% CI: 0.71–1.42, OR: 0.66, 95% CI: 0.30–1.41)] or overall survival [hazard ratio (HR): 1.01, 95% CI: 0.85–1.19, HR: 0.77, 95% CI: 0.52–1.14] in unmatched studies and RCT/adjusted studies. Late mortality (OR: 0.37, 95% CI: 0.21–0.64) and cardiac-related mortality (OR: 0.36, 95% CI: 0.21–0.62) were lower in the tricuspid valve repair (TVR) group in the RCT/adjusted studies. In the unmatched studies, overall cardiac mortality (OR: 0.48, 95% CI: 0.26–0.88) was lower in the TVR group. In the late TR progression analysis, the late TR progression was lower among patients in the concomitantly intervened tricuspid group, and patients in the untreated tricuspid group were prone to TR progression in both studies (HR: 0.30, 95% CI: 0.22–0.41, HR: 0.37, 95% CI: 0.23–0.58).

**Conclusions:**

TVR concomitant with MV surgery is most effective in patients with significant TR and dilated tricuspid annulus, especially those with a significantly reduced risk of distant TR progression.

## Introduction

Highlights

- Concomitant tricuspid valve repair was associated with an improved late prognosis, particularly a reduced risk of late mortality, cardiac-related mortality, and tricuspid regurgitation (TR) progression.
- Patients will benefit from concomitant tricuspid valve repair, and the results might be even more promising, especially in patients with significant TR and tricuspid valve dilatation.
- For significant TR, the reason for concomitant performance was that TR might not resolve after mitral valve surgery.
- Repair of TR associated with annular dilatation was done to prevent the worsening of tricuspid annular dilatation, or the development of severe TR.

The successful experience of undergoing mitral valve (MV) surgery for functional tricuspid regurgitation (FTR) was first reported in the 1950s^1^. In the 1960s, it was again demonstrated in a study by Braunwald *et al*.^2^ that FTR was resolved among patients with severe FTR who underwent MV replacement. Tricuspid regurgitation (TR) occurs in up to 64% of patients who undergo MV surgery for mitral regurgitation^3–6^. Carpentier *et al*.^7^ preference for routine tricuspid valve repair (TVR) for FTR, first described in the 1970s, also remains the prevailing view and it is accepted by most people, that severe TR may not improve effectively after MV surgery and should be addressed during MV surgery^8–10^. Although the American College of Cardiology/American Heart Association (ACC/AHA)^11^ and European Society of Cardiology/European Association for Cardio-Thoracic Surgery (ESC/EACTS)^12^ guidelines recommend that patients with severe TR should be treated with a TVR in conjunction with left ventricular surgery (Class I recommendation), and that symptomatic patients or right ventricular patients with severe enlargement be considered for tricuspid valve (TV) surgery in conjunction with left valve surgery (Class IIa recommendation), recommendations for managing TR during MV surgery are still largely based on observational data from clinical practice.

It has been proven in many studies that up to 74% of patients who undergo successful left-sided valve surgery will develop TR over time with poor outcomes^13–16^. The chief concern in the prognosis of patients with MV disease and concomitant TR include worsening of TR, impairment of right heart function, reduced quality of life, and consequently reduced life expectancy^11,12,17^. For this reason, many physicians believe that a more aggressive treatment and prophylactic approach to the TV is necessary alongside surgery in patients with concomitant MV disease concomitant TR^4,18,19^. A series of studies found that TVR was associated with less residual TR in the early postoperative period, and residual TR was associated with lower survival during follow-up^20–22^. Therefore, it is recommended that patients with FTR, particularly in the setting of a dilated tricuspid annulus, undergo concomitant TVR during MV surgery. However, Ro *et al.* ^23^ and Gammie *et al.* ^24^ suggested that there is no apparent significant advantage of TVR compared to patients who underwent MV surgery alone owing to risk factors such as age, diabetes, chronic renal failure, history of previous cardiac surgery, and concurrent Maze surgery for atrial fibrillation (AF). In addition, TVR leads to the more frequent implantation of permanent pacemakers^24^. Due to the scarcity of studies with rigorous long-term follow-up and insufficient follow-up data, uncertainties persist about whether to perform a combined valve procedure.

Although studies have been conducted on this issue over the past decades, reaching a unified conclusion has been challenging because scholars have varying understandings of MV disease secondary to TR. Whether TV should be repaired during MV surgery and the timing of prosthetic repair are debatable. This systematic review and meta-analysis was conducted to answer these questions by comparing clinical and follow-up data from patients who underwent TVR with or without concomitant surgery for MV disease.

## Review methods

### Registration and protocol

This systematic review and meta-analysis were conducted following the Preferred Reporting Items for Systematic Reviews and Meta-Analysis (PRISMA)^25^, Supplemental Digital Content 1, http://links.lww.com/JS9/A674, Supplemental Digital Content 6, http://links.lww.com/JS9/A679 and Assessing the methodological quality of systematic reviews (AMSTAR) guidelines^26^, Supplemental Digital Content 2, http://links.lww.com/JS9/A675. The protocol for this overview was registered on PROSPERO (CRD42022380967) and is accessible on the PROSPERO website (https://www.crd.york.ac.uk/prospero/), Supplemental Digital Content 3, http://links.lww.com/JS9/A676. Because this was an analysis of previously published data, no ethical approval was required.

### Search and study selection

A comprehensive search strategy was designed to search PubMed, Embase, Web of Science, the Cochrane Library, and the China National Knowledge Infrastructure (CNKI) and identify all relevant studies before May 2022. The search was performed by combining MeSH/Emtree *terms* and keywords. The retrieval process is shown in Supplementary Table 1, Supplemental Digital Content 4, http://links.lww.com/JS9/A677, using PubMed as an example.

### Eligibility criteria

The inclusion criteria were based on the PICO (population, intervention, control or comparator, and outcome) statement. All comparative studies were eligible for inclusion.

Clinical research question: Should TVR be performed during MV surgery?

PICO statement: P-patients, problem or population: Patients with MV disease and TR. I-intervention or exposure: TVR. C-comparison, control or comparator: To compare postoperative survival and TR in patients with and without TVR. O-outcome: The primary outcome indicators were 30-day mortality, late mortality, cardiac-related mortality, the odds of TVR as a risk factor for death, and freedom from late TR. The secondary outcome indicators were the grade of TR, stroke, pulmonary artery systolic pressure (PASP, mmHg), left ventricular ejection fraction (LVEF, %), cardiopulmonary bypass (CPB) time, and aorta cross-clamp (ACC) time.

### Data abstraction

The data extraction form was tested and revised before the extraction. Two researchers extracted and revised the data. If disagreements occurred during the extraction process, the decision was discussed with a third researcher. The main data extracted were: basic information about the study; basic patient characteristics and preoperative information; outcome indicators, including primary and secondary outcome indicators mentioned in the eligibility criteria.

In addition, two other issues required special attention. The first was the transformation between quartiles and SD of means, which we subscribed to using the online tool designed by Wan *et al.* ^27^. The second was the extraction of hazard ratios (HRs) from the survival curves, which we have done using the method of Tierney *et al.* ^28^.

### Quality assessment and risk of bias in individual studies

We assessed the risk of bias in randomized controlled trials (RCTs) using *The Cochrane Collaboration’s Tool for Risk of Bias* ^29^, which evaluates selection bias, performance bias, detection bias, attrition bias, and reporting bias. Each type of bias was rated as a ‘High’, ‘Low’, or ‘Unclear’ risk. For retrospective studies, the quality of each study was assessed with the *Newcastle–Ottawa Scale* ^30^ and scored based on population selection, comparability, and exposure to risk factors. Studies with scores greater than or equal to 7 were considered to be of high quality. This work was independently cross-checked with the original publications for accuracy and completeness by two other researchers. The results of the quality assessment were presented using Review Manager 5.4.

### Statistical analysis

Eleven analyses were performed using the open‐source R software version 4.1.3, accessed via the RStudio server. Dichotomous variables were presented as odds ratios (ORs) with 95% CIs, and continuous variables were presented as weighted mean differences or standardized mean differences (Std. MD) with 95% CIs. Additionally, HR was meta-analyzed using the inverse variance method. A *P-value* of 0.05 was applied as the cut-off for determining statistical significance. Statistical heterogeneity was assessed with the Cochran *Q-test* and *I* ^*2*^-*test*. If significant heterogeneity was observed (*I* ^*2*^ > 50% or *p*(Q) < 0.05), pooled estimates were calculated using a random‐effects model. Otherwise, a fixed‐effect model was used.

### Publication bias and sensitivity analyses

Publication bias of combined risk ratio estimates was evaluated by visual assessment funnel when the meta-analysis included more than 10 studies^31^, and asymmetry was assessed using Begg’s and Egger’s regression tests. Where there was moderate to high heterogeneity between studies, sensitivity analyses were conducted by sequentially omitting individual studies to determine the impact of the excluded studies on the pooled results.

## Results

### Literature selection and study characteristics

**Figure 1:**
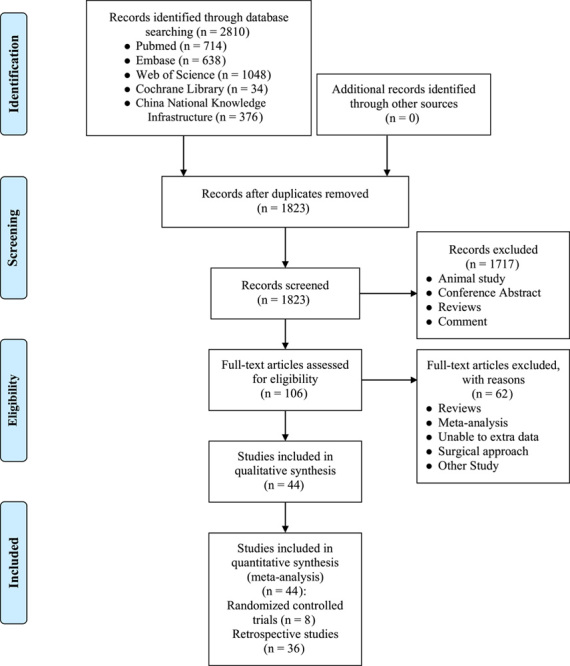
Flowchart showing the selection of potentially relevant studies for the present study.

We identified 2810 citations from five databases, and 1823 were reviewed for eligibility after removing duplicates. We ultimately included 44 studies^4,20–24,32–69^, of which eight^24,36,39,47,49–52^ were RCT studies and the rest were retrospective studies Figure 1. were RCTs, and the rest were retrospective studies Figure 1. The included studies were divided into three categories based on the degree of TR: studies with TR less than or equal to moderate (including mild, mild to moderate, and moderate); TR greater than or equal to moderate (including moderate-to-severe and severe); and studies with no classification of TR. During data extraction, we divided the data into unmatched and RCT/adjusted data categories, yielding 54 baseline data and 55 outcomes data. The studies by Hou *et al.* ^53^ and He *et al.* ^56^ classified different tricuspid valvuloplasty materials, so we included each of them as two separate studies. In the study by Zadok *et al.* ^55^, three comparisons were made because of the different levels of TR, so we also included their study as three separate studies. Supplementary Table 2, Supplemental Digital Content 4, http://links.lww.com/JS9/A677, shows baseline information for all included studies and patients.

### Quality assessment of individual studies

**Figure 2:**
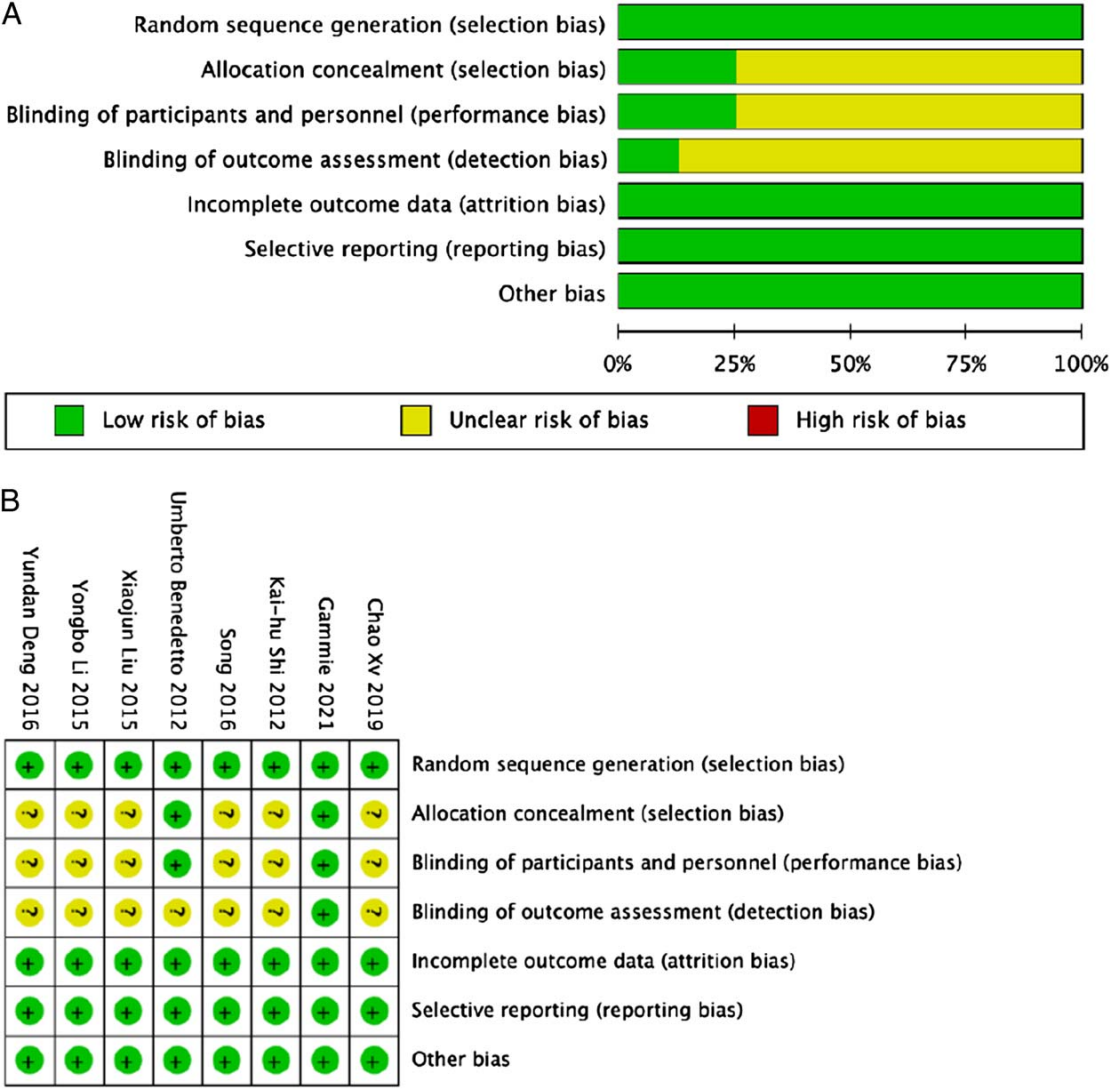
The quality assessment of RCTs. (A) Risk of bias graph: review authors’ judgments about each risk of bias item are presented as percentages across all included studies; (B) Risk of bias summary: review authors’ judgments about each risk of bias item for each included study.

**Figure 3:**
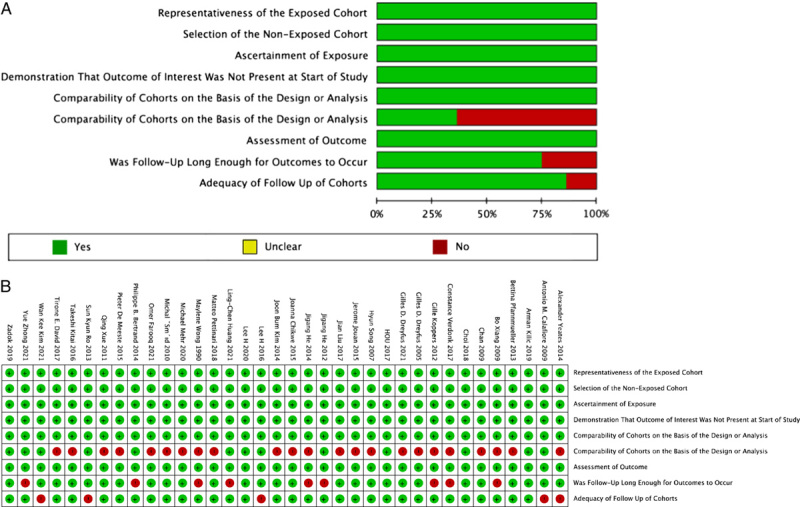
The quality assessment of retrospective studies. (A) Risk of bias graph: review authors’ judgments about each risk of bias item presented as percentages across all included studies; (B) Risk of bias summary: review authors’ judgments about each risk of bias item for each included study.

The results of the quality evaluation of the RCTs^24,36,39,47,49–52^ are presented in Figure 2. Generally, the risk of bias in the included studies was moderate to low and the quality of the literature was high. We ameliorated the description of allocation concealment, blinding of participants and personnel, and blinding of the outcome when assessing the quality of RCTs to ensure better research quality. The results of the *Newcastle–Ottawa Scale* scores of the retrospective studies^4,20–23,32–35,37,38,40–46,48,53–69^ are presented in Figure 3. In the comparability section, we gave a score of 2 if a study had a second grouping by controlling for impact factors. In the outcome section, our criteria for follow-up were a minimum of three years and a 95% follow-up rate, and we scored them separately if they met these criteria. In summary, all studies scored greater than or equal to 7 and were of good quality.

### Analysis of unmatched studies

#### Baseline characteristics and analysis

There were 32^4,20–23,32,35,38,40,41,43–46,48,53–69^ unmatched studies in total, with 34 separate extracts of baseline and outcome information. The TR was less than or equal to mild in six studies^21,22,41,48,68,69^, less than or equal to moderate in 13^20–23,38,40,41,48,53,54,67–69^, greater than or equal to moderate in eight^32,35,46,60,62–65^, and unclassified in the remainder of the studies. Baseline comparisons of the results showed that patients with TVR were slightly more complex than those without. First, patients in the TVR group were about a year older (MD: 1.40, 95% CI: 0.33–2.46) and were more likely to be women (OR: 1.24, 95% CI: 1.13–1.36) and have AF (OR: 2.54, 95% CI: 1.95–3.31) than those in the TVR group. In addition, the patients in the TVR group had a higher PASP (MD: 2.64, 95% CI: 0.79–4.49) and a longer TV annulus diameter (Std. MD: 0.65, 95% CI: 0.44–0.86) than those in the TVR group. Moreover, the TR and NYHA classifications were higher in the TVR group than in the TVR- group (P < 0.01). At baseline, the two groups did not differ by the following: diabetes mellitus, hypertension, stroke, coronary artery disease, and LVEF (P > 0.05). The above results are all presented in Table 1.

**Table 1:** Comparison of baseline information from unmatched studies.

				Participants								
Characteristics of the patients	Studies	Effect measure	Model	TVR	TVR-	Effect estimate	LCI	UCI	Q	*P* (Q)	Z	*P* (Z)
Age, years	33	Mean difference	Random	3692	5339	1.40	0.33	2.46	6.05	<0.01	2.58	<0.01
Female sex	34	Odds ratio	Fixed	3751	5415	1.24	1.13	1.36	0.06	<0.01	4.54	<0.01
Diabetes mellitus	15	Odds ratio	Fixed	2448	4140	0.97	0.81	1.16	0.00	0.50	–0.34	0.74
Hypertension	12	Odds ratio	Random	2143	3612	0.99	0.74	1.31	0.15	<0.01	–0.10	0.92
Stroke	8	Odds ratio	Fixed	1511	2487	0.97	0.73	1.28	0.00	0.44	–0.22	0.83
Coronary artery disease	8	Odds ratio	Fixed	878	1897	0.77	0.55	1.07	0.00	0.72	–1.57	0.12
Atrial fibrillation	25	Odds ratio	Random	3250	4817	2.54	1.95	3.31	0.30	<0.01	6.87	<0.01
PASP, mmHg	15	Mean difference	Random	1984	2507	2.64	0.79	4.49	8.62	<0.01	2.80	<0.01
LVEF, %	17	Mean difference	Random	2492	2845	–1.26	–2.64	0.13	6.30	<0.01	–1.78	0.08
TV annulus diameter	11	Std. mean difference	Random	1268	956	0.65	0.44	0.86	0.08	<0.01	6.04	<0.01
TR ≤ moderate	18	Odds ratio	Random	2605	4192	0.08	0.01	0.62	6.16	<0.01	–2.42	0.02
TR >moderate	10	Odds ratio	Random	1521	2240	33.87	10.11	113.52	1.68	<0.01	5.71	<0.01
NYHA ≤ 2	5	Odds ratio	Fixed	249	1379	0.43	0.30	0.60	0.16	0.13	–4.82	<0.01
NYHA > 2	21	Odds ratio	Random	1799	2926	1.49	1.14	1.95	0.23	<0.01	2.89	<0.01

LCI, low confidence interval; LVEF, left ventricular ejection fraction; PASP, pulmonary artery systolic pressure; TR, tricuspid regurgitation; TV, tricuspid valve; TVR, tricuspid valve repair; UCI, up confidence interval.

#### Primary outcomes

**Figure 4:**
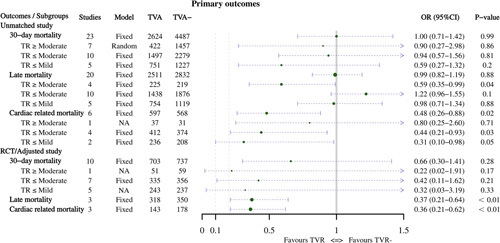
Summary forest plots of primary outcomes, including 30-day mortality, late mortality and cardiac-related mortality, were compared using odds ratio.

**Figure 5:**
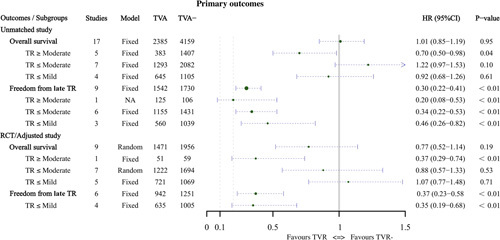
Summary forest plots of primary outcomes, including overall survival and freedom from late TR, were compared using hazard ratio.

In the analysis of 30-day mortality, there was no difference between the two groups in either the total combined value (OR: 1.00, 95% CI: 0.71–1.42, Supplementary Figure 1, Supplemental Digital Content 5, http://links.lww.com/JS9/A678) or the subgroup analysis according to TR (*P* > 0.05). For late mortality, the results were meaningful in the group with TR greater than or equal to moderate (OR: 0.59, 95% CI: 0.35–0.99, Supplementary Figure 2, Supplemental Digital Content 5, http://links.lww.com/JS9/A678). Compared to the TVR group, cardiac-related mortality was lower in the TVR group (OR: 0.48, 95% CI: 0.26–0.88, Supplementary Figure 3, Supplemental Digital Content 5, http://links.lww.com/JS9/A678), especially in the TR less than or equal to mild (OR: 0.31, 95% CI: 0.10–0.98) and TR less than or equal to moderate (OR: 0.44, 95% CI: 0.21–0.93) groups. Regarding the analysis of overall survival, TVR was favorable in patients with significant TR, and the results were statistically significant (HR: 0.70, 95% CI: 0.50–0.98, Supplementary Figure 4, Supplemental Digital Content 5, http://links.lww.com/JS9/A678). The results of freedom from late TR showed that concomitant TVR appears to be of great importance with a *P*-value of less than 0.05 for all groups (Supplementary Figure 5, Supplemental Digital Content 5, http://links.lww.com/JS9/A678). These results are presented in Table 2 and Figures 4–5.

**Table 2:** Results of a meta-analysis of unmatched studies primary outcome indicators.

				Participants								
Outcomes / Subgroups	Studies	Effect measure	Model	TVR	TVR-	Effect estimate	LCI	UCI	Q	*P* (Q)	Z	*P* (Z)
30-day mortality	23	Odds ratio	Fixed	2624	4487	1.00	0.71	1.42	0.15	0.23	0.01	0.99
TR ≥ Moderate	7	Odds ratio	Random	422	1457	0.90	0.27	2.98	1.15	0.04	–0.18	0.86
TR ≤ Moderate	10	Odds ratio	Fixed	1497	2279	0.94	0.57	1.56	0.00	0.48	–0.24	0.81
TR ≤ Mild	5	Odds ratio	Fixed	751	1227	0.59	0.27	1.32	0.00	0.99	–1.29	0.20
Late mortality	20	Odds ratio	Fixed	2511	2832	0.99	0.82	1.19	0.23	<0.01	–0.15	0.88
TR ≥ Moderate	4	Odds ratio	Fixed	225	219	0.59	0.35	0.99	0.32	0.16	–2.01	0.04
TR ≤ Moderate	10	Odds ratio	Fixed	1438	1876	1.22	0.96	1.55	0.18	0.05	1.66	0.10
TR ≤ Mild	5	Odds ratio	Fixed	754	1119	0.98	0.71	1.34	0.00	0.52	–0.15	0.88
Cardiac-related mortality	6	Odds ratio	Fixed	597	568	0.48	0.26	0.88	0.00	0.80	–2.37	0.02
TR ≥ moderate	1	Odds ratio	NA	37	31	0.80	0.25	2.60	NA	NA	–0.37	0.71
TR ≤ Moderate	4	Odds ratio	Fixed	412	374	0.44	0.21	0.93	0.00	0.78	–2.15	0.03
TR ≤ Mild	2	Odds ratio	Fixed	236	208	0.31	0.10	0.98	0.00	0.51	–1.99	0.05
Overall survival	17	Hazard ratio	Fixed	2385	4159	1.01	0.85	1.19	0.08	0.06	0.06	0.95
TR ≥ Moderate	5	Hazard ratio	Fixed	383	1407	0.70	0.50	0.98	0.00	0.47	–2.08	0.04
TR ≤ Moderate	7	Hazard ratio	Fixed	1293	2082	1.22	0.97	1.53	0.10	0.10	1.66	0.10
TR ≤ Mild	4	Hazard ratio	Fixed	645	1105	0.92	0.68	1.26	0.00	0.91	–0.51	0.61
Freedom from TR	9	Hazard ratio	Fixed	1542	1730	0.30	0.22	0.41	0.00	0.84	–7.44	<0.01
TR ≥ Moderate	1	Hazard ratio	NA	125	106	0.20	0.08	0.53	NA	NA	–3.24	<0.01
TR ≤ Moderate	6	Hazard ratio	Fixed	1155	1431	0.34	0.22	0.53	0.00	0.69	–4.81	<0.01
TR ≤ Mild	3	Hazard ratio	Fixed	560	1039	0.46	0.26	0.82	0.00	0.73	–2.62	<0.01

LCI, low confidence interval; TR, tricuspid regurgitation; TVR, tricuspid valve repair; UCI, up confidence interval.

#### Secondary outcomes

Results on the secondary outcomes are presented in Table 3. Patients in the postoperative TVR group had a significantly lower TR grade compared to baseline (TR ≤ moderate: 4.92 (3.65–6.64) versus 0.08 (0.01–0.62), Supplementary Figures 6–7, Supplemental Digital Content 5, http://links.lww.com/JS9/A678). In addition, the time for CPB (MD: 18.02, 95% CI: 8.59–27.45, Supplementary Figure 8, Supplemental Digital Content 5, http://links.lww.com/JS9/A678) and ACC (MD: 13.45, 95% CI: 7.61–19.29, Supplementary Figure 9, Supplemental Digital Content 5, http://links.lww.com/JS9/A678) were both much longer for patients in the TVR group than for those in the TVR group. Stroke, PASP, and LVEF were not found to be different between the two groups (P > 0.05, Supplementary Figures 10–12, Supplemental Digital Content 5, http://links.lww.com/JS9/A678).

**Table 3:** Results of a meta-analysis of unmatched studies’ and RCT/adjusted studies’ secondary outcome indicators.

				Participants								
Outcomes / Subgroups	Studies	Effect measure	Model	TVR	TVR-	Effect estimate	LCI	UCI	Q	*P* (Q)	Z	*P* (Z)
TR ≤ moderate												
RCT/Adjusted	9	Odds ratio	Fixed	379	403	0.38	0.23	0.64	0.00	0.63	–3.64	<0.01
Unmatched	12	Odds ratio	Random	1044	1045	0.25	0.06	0.98	4.17	<0.01	–1.99	<0.01
TR ≥ moderate												
RCT/Adjusted	9	Odds ratio	Fixed	379	403	4.47	2.90	6.88	0.00	0.99	6.80	<0.01
Unmatched	12	Odds ratio	Fixed	1044	1045	4.92	3.65	6.64	0.00	0.87	10.47	<0.01
CPB, time												
RCT/Adjusted	11	Mean difference	Random	651	652	19.11	11.05	27.16	105.77	<0.01	4.65	<0.01
Unmatched	13	Mean difference	Random	1720	2074	18.02	8.59	27.45	249.09	<0.01	3.74	<0.01
ACC, time												
RCT/Adjusted	11	Mean difference	Random	651	652	12.30	6.06	18.54	43.37	0.03	3.86	<0.01
Unmatched	13	Mean difference	Random	1720	2074	13.45	7.61	19.29	91.10	<0.01	4.51	<0.01
Stroke												
RCT/Adjusted	3	Odds ratio	Fixed	231	231	1.00	0.20	5.02	0.00	1.00	0.00	1.00
Unmatched	6	Odds ratio	Fixed	989	2229	0.88	0.56	1.36	0.00	0.74	–0.59	0.56
PASP, mmHg												
RCT/Adjusted	5	Mean difference	Random	245	242	0.38	–3.41	4.17	13.05	<0.01	0.19	0.85
Unmatched	6	Mean difference	Random	482	514	–0.10	–2.98	2.77	10.46	<0.01	–0.07	0.94
LVEF, %												
RCT/Adjusted	5	Mean difference	Fixed	143	131	–0.96	–3.03	1.10	0.00	0.91	–0.92	0.36
Unmatched	8	Mean difference	Fixed	363	331	0.38	–0.56	1.32	2.25	0.09	0.79	0.43

ACC, aorta cross-clamp; CPB, cardiopulmonary bypass; LCI, low confidence interval; LVEF, left ventricular ejection fraction; PASP, pulmonary artery systolic pressure; RCT, tricuspid valve; TR, tricuspid regurgitation; UCI, up confidence interval.

### RCT/adjusted study analysis

#### Baseline characteristics and analysis

Eighteen^21–24,33,34,36,37,39,41,42,47–52,55^ RCT/adjusted studies were included in this meta-analysis, including 20 outcome data. Baseline comparisons showed that more patients in the TVR group had AF (OR: 1.73, 95% CI: 1.14–2.62) and TR > moderate (OR: 31.37, 95% CI: 12.60–78.06) than those in the TVR group. In addition, the TV annulus diameter appeared to be a little longer in the TVR group (Std. MD: 0.51, 95% CI: 0.16–0.86). No other significant baseline differences (P > 0.05) were observed between the two groups (Table 4).

**Table 4:** Comparison of baseline information from RCT/adjusted studies.

				Participants								
Characteristics of the patients	Studies	Effect measure	Model	TVR	TVR-	Effect Estimate	LCI	UCI	Q	*P* (Q)	Z	*P* (Z)
Age, years	19	Mean difference	Fixed	1558	1937	–0.43	–1.14	0.29	0.00	0.96	–1.16	0.25
Female sex	17	Odds ratio	Fixed	1420	1790	1.00	0.86	1.16	0.00	0.63	–0.06	0.95
Diabetes mellitus	12	Odds ratio	Random	1166	1546	0.92	0.56	1.51	0.52	<0.01	–0.32	0.75
Hypertension	10	Odds ratio	Fixed	1145	1486	0.98	0.82	1.17	0.00	0.95	–0.22	0.82
Coronary artery disease	3	Odds ratio	Random	257	266	0.71	0.11	4.51	2.49	<0.01	–0.37	0.71
Atrial fibrillation	14	Odds ratio	Random	1201	1577	1.73	1.14	2.62	0.40	<0.01	–2.58	<0.01
PASP, mmHg	10	Mean difference	Fixed	907	1303	0.45	–0.213	1.11	1.45	0.09	1.33	0.18
LVEF, %	12	Mean difference	Fixed	1078	1446	0.06	–0.65	0.76	0.40	0.26	0.16	0.88
TV annulus diameter	8	Std. Mean difference	Random	560	540	0.51	0.16	0.86	0.21	<0.01	2.83	<0.01
TR ≤ moderate	13	Odds ratio	Random	1134	1613	0.24	0.04	1.25	2.59	<0.01	–1.69	0.09
TR > moderate	3	Odds ratio	Fixed	279	221	31.37	12.60	78.06	0.15	0.27	7.41	<0.01
NYHA ≤ 2	4	Odds ratio	Fixed	279	288	0.669	0.44	1.02	0.00	0.62	–1.88	0.06
NYHA > 2	14	Odds Ratio	Fixed	933	919	0.88	0.70	1.10	0.00	0.65	-1.14	0.25

LCI, low confidence interval; LVEF, left ventricular ejection fraction; PASP, pulmonary artery systolic pressure; TR, tricuspid regurgitation; TV, tricuspid valve; TVR, tricuspid valve repair; UCI, up confidence interval.

#### Primary outcomes

The results for the primary outcomes are presented in Table 5 and Figures 4–5. In the RCT/adjusted studies, there were no significant differences in the 30-day mortality (OR: 0.66, 95% CI: 0.30–1.41, Supplementary Figure 13, Supplemental Digital Content 5, http://links.lww.com/JS9/A678) and overall survival (HR: 0.77, 95% CI: 0.52–1.14, Supplementary Figure 14, Supplemental Digital Content 5, http://links.lww.com/JS9/A678) between the two groups. In the subgroup analysis categorized by TR class, the TR greater than or equal to moderate group had significant results despite having only one study (HR: 0.37, 95% CI: 0.19–0.74). Nevertheless, none of the other subgroup analyses for these two outcomes was significant.

**Table 5:** Results of a meta-analysis of RCT/adjusted studies primary outcome indicators.

				Participants								
Outcomes / Subgroups	Studies	Effect measure	Model	TVR	TVR-	Effect estimate	LCI	UCI	Q	*P* (Q)	Z	*P* (Z)
30-day mortality	10	Odds ratio	Fixed	703	737	0.66	0.30	1.41	0.00	0.61	–1.08	0.28
TR ≥ Moderate	1	Odds ratio	NA	51	59	0.22	0.02	1.91	NA	NA	–1.38	0.17
TR ≤ Moderate	7	Odds ratio	Fixed	335	356	0.42	0.11	1.62	0.00	0.80	–1.26	0.21
TR ≤ Mild	5	Odds ratio	NA	243	237	0.32	0.03	3.19	NA	NA	–0.97	0.33
Late mortality	3	Odds ratio	Fixed	318	350	0.37	0.21	0.64	0.16	0.24	–3.56	<0.01
Cardiac-related mortality	3	Odds ratio	Fixed	143	178	0.36	0.21	0.62	0.00	0.41	–3.74	<0.01
Overall survival	9	Hazard ratio	Random	1471	1956	0.77	0.52	1.14	0.20	0.01	–1.32	0.19
TR ≥ Moderate	1	Hazard ratio	NA	51	59	0.37	0.19	0.74	NA	NA	–2.81	<0.01
TR ≤ Moderate	7	Hazard ratio	Random	1222	1694	0.88	0.57	1.33	0.16	0.04	–0.62	0.53
TR ≤ Mild	5	Hazard ratio	Fixed	721	1069	1.07	0.77	1.48	0.00	0.79	0.38	0.71
Freedom from TR	6	Hazard ratio	Fixed	942	1251	0.37	0.23	0.58	<0.01	0.42	–4.31	<0.01
TR ≤ Mild	4	Hazard ratio	Fixed	635	1005	0.35	0.19	0.68	0.00	0.39	–3.13	<0.01

LCI, low confidence interval; TR, tricuspid regurgitation; TVR, tricuspid valve repair; UCI, up confidence interval.

Notably, the analysis of both late mortality (OR: 0.37, 95% CI: 0.21–0.64, Supplementary Figure 15, Supplemental Digital Content 5, http://links.lww.com/JS9/A678) and cardiac-related mortality (OR: 0.36, 95% CI: 0.21–0.62, Supplementary Figure 16, Supplemental Digital Content 5, http://links.lww.com/JS9/A678) showed that the TVR group had significantly better survival. In addition, the results of freedom from late TR, which was a key endpoint, remained significantly different (HR: 0.37, 95% CI: 0.23–0.58, Supplementary Figure 17, Supplemental Digital Content 5, http://links.lww.com/JS9/A678), suggesting a significant effect of TVR, especially in the group of TR less than or equal to mild.

#### Secondary outcomes

Secondary outcome results were similar to the unmatched studies. More patients in the TVR group converted to TR less than or equal to moderate, and the number of high-grade TR decreased (TR ≤ moderate: 4.47(2.90–6.88) versus 0.24(0.04–1.25), Supplementary Figures 6–7, Supplemental Digital Content 5, http://links.lww.com/JS9/A678), which was accompanied by longer CPB (MD: 19.11, 95% CI: 11.05–27.16, Supplementary Figure 8, Supplemental Digital Content 5, http://links.lww.com/JS9/A678) and ACC (MD: 12.30, 95% CI: 6.06–18.54, Supplementary Figure 9, Supplemental Digital Content 5, http://links.lww.com/JS9/A678) times. Stroke, PASP, and LVEF remained statistically insignificant (Supplementary Figures 10–12, Supplemental Digital Content 5, http://links.lww.com/JS9/A678). The results of the secondary outcomes are presented in Table 3.

### Publication bias and sensitivity analyses

**Figure 6:**
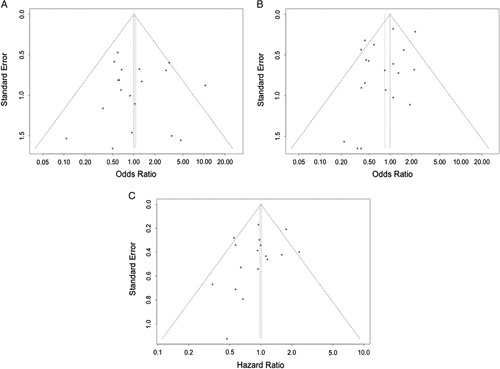
A collection of funnel plots for unmatched studies. (A) 30-day mortality; (B) late mortality; (C) overall survival.

**Figure 7:**
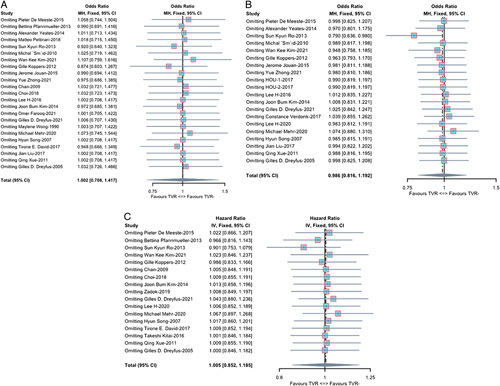
A collection of sensitivity analyses of unmatched studies. (A) 30-day mortality; (B) late mortality; (C) overall survival.

As stated in the methodology, tests for publication bias are only reliable when the included literature is greater than 10. Therefore, we performed tests for publication bias for 30-day mortality, late mortality, and overall survival in unmatched studies. The funnel plots are shown in Figure 6. The Begg’s and Egger’s regression tests showed no publication bias, with *P* values greater than 0.05. In sensitivity analyses, the results of the primary outcome indicators were robust and did not change after excluding a particular study (Fig. 7).

## Discussion

FTR is mostly secondary to functional regurgitation due to an enlarged right ventricle and dilated tricuspid annulus without significant organic pathology of the valve itself^3,6,70^. It is often secondary to disease of the left heart system, particularly MV disease^5,71^. In the case of FTR, which is progressive, surgical treatment of left heart systemic valve disease alone did not adequately resolve or prevent TV insufficiency, this was especially true in persistent pulmonary hypertension. Patients with severe TR may have no obvious clinical symptoms in the early stages and are not given sufficient attention by patients and physicians, often missing the best time to operate when surgery is recommended, which is a key reason for the high mortality rate of TR surgery^72^. However, active and effective intervention can improve the prognosis of patients^24,65^. Compared with previously published meta-analyses^73–76^, the chief advantages of this study are that it used a more comprehensive analysis of the prognosis of concomitantly managing different levels of TR during MV surgery and combined the unmatched studies and the RCT/adjusted studies in separate analyses to provide cardiac surgeons and cardiologists worldwide with better evidence-based solutions to this challenge.

In the baseline comparison, we found that more people in the TVR group had a larger tricuspid annulus and more AF compared to the TVR group in both the unmatched and RCT/adjusted studies. Chan *et al.* ^46^ suggested more aggressive treatment of TV in patients with TR secondary to MV surgery and a dilated tricuspid annulus during clinical procedures. It is worth noting that surgery should not be performed in patients who have only a dilated tricuspid annulus and no significant TR. This view was also more widely accepted^20,46,58^. In a prospective study of over 300 patients, Dreyfus *et al*.^4^ observed that remodeling annuloplasty based on tricuspid annular dilatation improved their functional status, regardless of the degree of preoperative TR. Dilation of the tricuspid annulus might occur even without significant TR. Virtually all of this is true regarding the pathogenesis of FTR, where tricuspid annular dilatation was predominant, and tricuspid annular size may be more reliable than its degree of regurgitation in predicting long-term prognosis. This is because the dilatation of the tricuspid annulus was objectively measurable, whereas TR could vary with cardiac preload, afterload, and right ventricular function. Looking at some postoperative outcomes, longer CPB and ACC times did not appear to have made much difference in the perioperative period but benefited patients in the long-term. The lower late mortality and cardiac mortality in the group with concomitant TV surgery was a very visual statistic.

In the analysis of overall survival, survival was better in patients with moderate-to-severe TR who received TVR, regardless of whether the studies were unmatched or RCT/adjusted. However, this seems to be widely accepted, and we are more interested in knowing whether there are positive outcomes for patients with mild or mild to moderate TR who receive TVR. This also suggests a direction for follow-up studies. The results of this meta-analysis, including subgroup analysis of TR as an independent risk factor for the poorer long-term prognosis of patients, showed that TR progression was less likely to occur in patients who underwent TVR, especially for the moderate-to-severe TR. This is in line with what we had hoped for and indicates that interventions for TV were meaningful. Clinical experience has shown that with a successful MV surgery, severe postoperative residual TR contributes to poor postoperative prognosis^13–16^. King *et al.* ^77^ studied patients who required a repeat TV procedure after MV surgery and observed that they had high early and late mortality rates. Therefore, the authors of this article encourage a strategy of concomitant undifferentiated tricuspid valvuloplasty during MV surgery. Surgical results have demonstrated that successful TVR, when combined with other valvular procedures, significantly reduces recurrent TR and improves survival. Gammie *et al.* ^24^ showed that despite a higher rate of progression to severe TR in the TVR group, no significant differences in symptoms and quality of life were observed between the two groups at 2-year follow-up, suggesting that aggressive TVR treatment may not be necessary for high-risk patients with limited life expectancy, as it did not result in symptomatic and quality of life improvements. This suggested that aggressive TVR treatment may not be necessary for patients with limited life expectancy, as it did not improve symptoms and quality of life. In addition, mild FTR in degenerative MR was reported in a 16-year study as being unlikely to progress^21^. A preventive TVR for mild TR may not benefit TR progression in degenerative MR. In addition, in the subgroup analysis, we found that the TVR group did not perform well in the analysis of late mortality (OR: 1.19, 95% CI: 0.94–1.51) and overall survival (HR: 1.21, 95% CI: 0.97–1.52) in the group with TR less than or equal to 2. This was a further reminder that although aggressive and effective TVR could benefit patients, more care should be taken when performing MV surgery in conjunction with TVR, especially for mild and mild to moderate TR.

MV surgery with TVR is done to avoid the progression of TR and the potential risk of future TV repair or replacement. However, the in-hospital mortality rate for TVR reoperations has been reported to be over 13%^20^. In addition, choosing an appropriate and effective repair has become an important issue. Currently, the main approaches to TVR are suture annuloplasty and prosthetic tricuspid annuloplasty^78–80^. Suture annuloplasties, such as the Kay method^81^ and the De Vega method^82^, have the advantage of technical simplicity and a low financial burden on the patient. However, the Kay method diastases TV and had a high rate of long-term postoperative regurgitation^17^, while the De Vega lacked strong support and reinforcement of the tricuspid annulus^83^, and both have unsatisfactory long-term results. In contrast, prosthetic tricuspid annuloplasty can better restore the TV^84,85^, particularly with its 3D rigid annuloplasty, which offers good early advantages^86,87^. In addition, transcatheter TV interventions offer a new way of thinking about FTR^88^. The latest ESC/EACTS provided the first recommendation for interventional treatment of TR in patients with symptomatic secondary TR who are unable to undergo surgery; interventional treatment of TV disease should be considered in the context of a discussion with heart valve center specialists (Class IIb recommendation)^12^. In recent years, studies in several centers have shown that TV interventional techniques were safe and feasible and could reduce the degree of regurgitation and improve the patient’s heart failure symptoms after the procedure^89^. However, longer follow-ups and future RCT studies of more patients are needed to evaluate these transcatheter TV repair techniques in this patient cohort before any real change can be made. For this reason, we believe that choosing the right approach at the right time to deal with TR during MV surgery would maximize the benefit to the patient.

## Limitation

The results of this meta-analysis must be interpreted with caution in the context of some significant limitations. First, the only eight RCTs we included in this study were more retrospectives in comparison. In addition, most of the studies did not use statistical techniques to adjust for potential differences in baseline demographics, which may have led to more heterogeneity. Although, we analyzed data from the RCT/adjusted and unmatched studies separately, we recognize that even with sophisticated statistical techniques, such as propensity score matching, our findings had unknown confounding factors. In particular, the patient’s presentation before the procedure, the surgeon’s understanding of the surgical expertise, and the completeness of the follow-up. Second, although the question of whether to manage the TV concomitantly was described in the ACC/AHA and ESC/EACTS guidelines, there was no strict criterion and the decision to operate concomitantly in most studies was based on the grade of the TR, whether the tricuspid annulus was dilated, the patient’s clinical symptoms and ultimately the surgeon’s decision. This would seem to leave comparability between patients open to concern. Although, we performed a comparability analysis of baseline information, the effect of confounding factors could not be avoided. Third, there was some bias in the extraction of data for HR from survival curves. While the data we extracted were consistent with the significance of the data reported in the article, it was still a secondary extraction performed by a machine or manually and is susceptible to bias.

## Conclusion

Concomitant TVR was associated with an improved late prognosis, particularly a reduced risk of late mortality, cardiac-related mortality, and TR progression. Patients will benefit from concomitant TVR, and the results may be even more promising, especially in patients with significant TR and TV dilatation. However, the single dilated tricuspid annulus should not be used as an indication for surgery. For significant TR, the reason for concomitant performance was that TR might not resolve after MV surgery. Repair of TR associated with annular dilatation was performed to prevent the worsening of tricuspid annular dilatation or the development of severe TR. If validated by forthcoming larger clinical trials, these results would certainly prompt a change in current guidelines for a more aggressive and effective approach to treating FTR during MV surgery.

## Ethics approval and consent to participate

Not applicable.

## Consent for publication

Written informed consent for publication was obtained from all participants.

## Sources of funding

Natural Science Foundation of Gansu Province (22JR5RA655, 21JR1RA027).

## Author contribution

K.Y., W.W., and T.Y.: wrote the main manuscript text; J.X., X.Z., W.W., C.L., and X.L.: prepared figures and tables. All authors reviewed the manuscript.

## Conflicts of interest disclosure

The authors declared that there have no conflicts of interest in this work.

## Research registration unique identifying number (UIN)

PROSPERO, identifier: CRD42022380967.

## Guarantor

Kang Yi, Wei Wang and Tao You.

## Data availability statement

All data generated or analyzed during this study are included in this published article.

## Provenance and peer review

Not commissioned, externally peer-reviewed.

## Supplementary Material
