# Supplementary material for: Tricuspid valve repair concomitant with mitral valve surgery: a systematic review and meta-analysis
Source: Int J Surg. 2023 Jun 7;109(7):2082–95. doi: 10.1097/JS9.0000000000000396 (PMC10389546; doi:10.1097/JS9.0000000000000396)
Supplement: Supplementary file 3 [file js9-109-2082-s003.pdf]

Systematic review

A list of fields that can be edited in an update can be found here

1. * Review title.

Give the title of the review in English

Tricuspid valve repair concomitant with mitral valve surgery: a systematic review and meta?analysis

2. Original language title.

For reviews in languages other than English, give the title in the original language. This will be displayed with the English language title.

Tricuspid valve repair concomitant with mitral valve surgery: a systematic review and meta?analysis

3. * Anticipated or actual start date.

Give the date the systematic review started or is expected to start.

03/05/2022

4. * Anticipated completion date.

Give the date by which the review is expected to be completed.

04/01/2023

5. * Stage of review at time of this submission. [1 change]

This field uses answers to initial screening questions. It cannot be edited until after registration.

Tick the boxes to show which review tasks have been started and which have been completed.

Update this field each time any amendments are made to a published record.

The review has not yet started: No

Review stage	Started	Completed
Preliminary searches	Yes	Yes
Piloting of the study selection process	Yes	Yes
Formal screening of search results against eligibility criteria	Yes	Yes
Data extraction	Yes	Yes
Risk of bias (quality) assessment	Yes	Yes
Data analysis	Yes	Yes

Provide any other relevant information about the stage of the review here.

6. * Named contact.

The named contact is the guarantor for the accuracy of the information in the register record. This may be any member of the review team.

wei wang

Email salutation (e.g. "Dr Smith" or "Joanne") for correspondence:

Dr wang

7. * Named contact email.

Give the electronic email address of the named contact.

wangw20@126.com

8. Named contact address

Give the full institutional/organisational postal address for the named contact.

Department of Cardiac Surgery, The First Hospital of China Medical University, Shenyang, Liaoning, China.

9. Named contact phone number.

Give the telephone number for the named contact, including international dialling code.

15379317186

10. * Organisational affiliation of the review.

Full title of the organisational affiliations for this review and website address if available. This field may be

completed as 'None' if the review is not affiliated to any organisation.

Department of Cardiac Surgery, The First Hospital of China Medical University, Shenyang, Liaoning, China.

Organisation web address:

11. * Review team members and their organisational affiliations.

Give the personal details and the organisational affiliations of each member of the review team. Affiliation refers to groups or organisations to which review team members belong. **NOTE: email and country now MUST be entered for each person, unless you are amending a published record.**

Dr Kang Yi. Department of Cardiovascular Surgery, Gansu Provincial Hospital, Lanzhou, Gansu Province, China.
Dr Wei Wang. Department of Cardiac Surgery, The First Hospital of China Medical University, Shenyang, Liaoning, China.
Jianguo Xu. Evidence-Based Medicine Center, School of Basic Medical Sciences, Lanzhou University, Lanzhou, China.
Xin Zhang. The First School of Clinical Medicine of Gansu University of Chinese Medicine, Lanzhou, China.
Wenxin Wang. The First School of Clinical Medicine of Gansu University of Chinese Medicine, Lanzhou, China
Chengfei Liu. The First Clinical Medical College of Lanzhou University, Lanzhou, China.
Xinyao Li. Shengjing Hospital of China Medical University, Shenyang, Liaoning, China.
Tao You. Department of Cardiovascular Surgery, Gansu Provincial Hospital, Lanzhou, Gansu Province, China.

12. * Funding sources/sponsors.

Details of the individuals, organizations, groups, companies or other legal entities who have funded or sponsored the review.

Natural Science Foundation of Gansu Province (22JR5RA655, 21JR1RA027)

Grant number(s)

State the funder, grant or award number and the date of award

13. * Conflicts of interest.

List actual or perceived conflicts of interest (financial or academic).

None

14. Collaborators.

Give the name and affiliation of any individuals or organisations who are working on the review but who are not listed as review team members. **NOTE: email and country must be completed for each person, unless you are amending a published record.**

15. * Review question.

State the review question(s) clearly and precisely. It may be appropriate to break very broad questions down into a series of related more specific questions. Questions may be framed or refined using PI(E)COS or similar where relevant.

The question of whether to intervene aggressively and effectively in tricuspid regurgitation (TR) in conjunction with mitral valve (MV) surgery has long been debated. With this background in mind, we have prepared this systematic review and meta-analysis to provide answers to these questions by comparing clinical and follow-up data from patients who underwent concomitant TVR with or without surgery for MV disease.

16. * Searches.

State the sources that will be searched (e.g. Medline). Give the search dates, and any restrictions (e.g. language or publication date). Do NOT enter the full search strategy (it may be provided as a link or attachment below.)

A comprehensive search strategy designed to allow exhaustive identification of relevant studies was applied to the PubMed, Embase, Web of Science, Cochrane Library and China National Knowledge Infrastructure (CNKI) before May 2022. The literature search was performed using a combination of MeSH/Emtree terms and keywords.

17. URL to search strategy.

Upload a file with your search strategy, or an example of a search strategy for a specific database, (including the keywords) in pdf or word format. In doing so you are consenting to the file being made publicly accessible. Or provide a URL or link to the strategy. Do NOT provide links to your search **results**.

SET QUERY

#1 Mitral Valve Annuloplasty [Mesh]

#2 Mitral Valve Annuloplasties

#3 Mitral Annuloplasty

#4 Mitral Annuloplasties

#5 Mitral Valve Annulus Repair

#6 Mitral Valvuloplasty

#7 Mitral valve repair

#8 Mitral valve surgery

#9 Mitral valve replacement

#10 #1 OR #2 OR #3 OR #4 OR #5 OR #6 OR #7 OR #8 OR #9 OR #10

#11 tricuspid valve insufficiency [Mesh]

#12 tricuspid valve insufficiency

#13 Tricuspid Valve Incompetence

#14 Tricuspid Incompetence

#15 functional tricuspid [Mesh]

#16 functional tricuspid

#17 tricuspid Valve Regurgitation

#18 functional tricuspid regurgitation

#19 tricuspid regurgitation

#20 isolated tricuspid regurgitation

#21 isolated tricuspid valve regurgitation

#22 isolated tricuspid valve incompetence

#23 Congenital tricuspid regurgitation

#24 Isolated congenital tricuspid regurgitation

#25 #11 OR #12 OR #13 OR #14 OR #15 OR #16 OR #17 OR #18 OR#19 OR #20 OR #21 OR #22 OR #23 OR #24

#26 #10 AND #25

Alternatively, upload your search strategy to CRD in pdf format. Please note that by doing so you are consenting to the file being made publicly accessible.

Do not make this file publicly available until the review is complete

18. * Condition or domain being studied.

Give a short description of the disease, condition or healthcare domain being studied in your systematic review.

The question of whether to intervene aggressively and effectively in tricuspid regurgitation (TR) in conjunction with mitral valve (MV) surgery has long been debated. Although different scholars have worked

on this issue differently over the past decades, reaching a unified conclusion has not been possible because each scholar has a different understanding of MV disease secondary to TR. The question of whether we should repair the TV while undergoing MV surgery, and the timing of prosthetic repair, is still open to debate. With this background in mind, we have prepared this systematic review and meta-analysis to provide answers to these questions by comparing clinical and follow-up data from patients who underwent concomitant TVR with or without surgery for MV disease.

19. * Participants/population.

Specify the participants or populations being studied in the review. The preferred format includes details of both inclusion and exclusion criteria.

Patients with MV disease with tricuspid regurgitation.

20. * Intervention(s), exposure(s).

Give full and clear descriptions or definitions of the interventions or the exposures to be reviewed. The preferred format includes details of both inclusion and exclusion criteria.

tricuspid valve repair

21. * Comparator(s)/control.

Where relevant, give details of the alternatives against which the intervention/exposure will be compared (e.g. another intervention or a non-exposed control group). The preferred format includes details of both inclusion and exclusion criteria.

To compare postoperative survival and tricuspid regurgitation in patients with and without TVR.

22. * Types of study to be included.

Give details of the study designs (e.g. RCT) that are eligible for inclusion in the review. The preferred format includes both inclusion and exclusion criteria. If there are no restrictions on the types of study, this should be stated.

RCT studies and retrospective studies

23. Context.

Give summary details of the setting or other relevant characteristics, which help define the inclusion or exclusion criteria.

24. * Main outcome(s).

Give the pre-specified main (most important) outcomes of the review, including details of how the outcome is defined and measured and when these measurement are made, if these are part of the review inclusion criteria.

The primary outcome indicators were 30-day mortality, late mortality, cardiac-related mortality, the odds of TVR as a risk factor for death and the freedom from late TR.

Measures of effect

Please specify the effect measure(s) for you main outcome(s) e.g. relative risks, odds ratios, risk difference, and/or 'number needed to treat.

25. * Additional outcome(s).

List the pre-specified additional outcomes of the review, with a similar level of detail to that required for main outcomes. Where there are no additional outcomes please state 'None' or 'Not applicable' as appropriate to the review

The secondary outcome indicators were the grade of TR, stroke, pulmonary artery systolic pressure (PASP, mmHg), left ventricular ejection fraction (LVEF, %), cardiopulmonary bypass (CPB) time and aorta cross-clamp (ACC) time.

Measures of effect

Please specify the effect measure(s) for you additional outcome(s) e.g. relative risks, odds ratios, risk difference, and/or 'number needed to treat.

26. * Data extraction (selection and coding).

Describe how studies will be selected for inclusion. State what data will be extracted or obtained. State how this will be done and recorded.

The data extraction was extracted and revised by two researchers together, and the data extraction form was tested and refined before starting the extraction. If disagreements occurred during the extraction process, the decision was discussed with a third researcher. The main data extracted were as follows:(1) Basic information about the study. (2) Basic patient characteristics and preoperative information. (3) Outcome Indicators, included primary and secondary outcome indicators in the methods for the eligibility criteria. In addition, two other issues require special attention. The first is the transformation between quartiles and standard deviations of means, which we subscribed to using the online tool designed by Wan et al[1]. The second is the extraction of hazard ratios from the survival curves, which we have done using the method of Tierney et al[2].

27. * Risk of bias (quality) assessment.

State which characteristics of the studies will be assessed and/or any formal risk of bias/quality assessment tools that will be used.

We assessed the risk of bias in Randomized Controlled Trials (RCTs) using The Cochrane Collaboration's Tool for Risk of Bias[3], which reported selection bias, performance bias, detection bias, attrition bias and reporting bias. Each characteristic of bias was rated as a "High", "Low" or "Unclear" risk. For retrospective studies, the quality of each study was assessed with the Newcastle-Ottawa Scale (NOS)[4], which was scoring standard from three aspects: population selection, comparability, and exposure to risk factors. It was generally believed that the research was considered high-quality when the score was ?7. This work was

independently cross-checked="checked" value="1" with the original publications for accuracy and completeness by two other researchers. The results of the evaluation were presented using Review Manager 5.4.

28. * Strategy for data synthesis.

Describe the methods you plan to use to synthesise data. This **must not be generic text** but should be **specific to your review** and describe how the proposed approach will be applied to your data. If meta-analysis is planned, describe the models to be used, methods to explore statistical heterogeneity, and software package to be used.

All analysis was performed using the open?source R software accessed via the RStudio server (R version 4.1.3). The dichotomous variables are presented as odds ratios (ORs) with 95% CIs, and continuous variables are presented as weighted mean differences [MD] or standardized mean differences (Std. MD) with 95% CIs. Additionally, HR was subjected to meta-analysis using the inverse variance method. A P-value of 0.05 was applied as the cut-off for determining statistical significance. Statistical heterogeneity was assessed with the Cochran Q-test and I^2-test. If significant heterogeneity was observed (I^2 50% or p(Q)?0.05), pooled estimates were calculated using a random?effects model, otherwise a fixed?effects model was used.

29. * Analysis of subgroups or subsets.

State any planned investigation of ‘subgroups’. Be clear and specific about which type of study or participant will be included in each group or covariate investigated. State the planned analytic approach.

Visual assessment funnel plots assessed publication bias of combined risk ratio estimates when the meta-analysis included more than 10 studies[5], and asymmetry was assessed using Begg’s and Egger’s regression tests. Where there was moderate to high heterogeneity between studies, sensitivity analyses were conducted by sequentially omitting individual studies to determine the impact of the studies on the pooled results.

30. * Type and method of review.

Select the type of review, review method and health area from the lists below.

Type of review

Cost effectiveness
No

Diagnostic
No

Epidemiologic
No

Individual patient data (IPD) meta-analysis
No

Intervention

Yes

Living systematic review

No

Meta-analysis

Yes

Methodology

No

Narrative synthesis

No

Network meta-analysis

No

Pre-clinical

No

Prevention

No

Prognostic

Yes

Prospective meta-analysis (PMA)

No

Review of reviews

No

Service delivery

No

Synthesis of qualitative studies

No

Systematic review

Yes

Other

No

Health area of the review

Alcohol/substance misuse/abuse

No

Blood and immune system

No

Cancer

No

Cardiovascular

Yes

Care of the elderly

No

Child health

No

Complementary therapies

No

COVID-19

No

Crime and justice

No

Dental

No

Digestive system

No

Ear, nose and throat

No

Education

No

Endocrine and metabolic disorders

No

Eye disorders

No

General interest

No

Genetics

No

Health inequalities/health equity

No

Infections and infestations

No

International development

No

Mental health and behavioural conditions

No

Musculoskeletal

No

Neurological

No

Nursing

No

Obstetrics and gynaecology

No

Oral health

No

Palliative care

No

Perioperative care

No

Physiotherapy

No

Pregnancy and childbirth

No

Public health (including social determinants of health)

No

Rehabilitation

No

Respiratory disorders

No

Service delivery

No

Skin disorders

No

Social care

No

Surgery

Yes

Tropical Medicine

No

Urological

No

Wounds, injuries and accidents

No

Violence and abuse

No

31. Language.

Select each language individually to add it to the list below, use the bin icon to remove any added in error.

English

There is not an English language summary

32. * Country.

Select the country in which the review is being carried out. For multi-national collaborations select all the countries involved.

China

33. Other registration details.

Name any other organisation where the systematic review title or protocol is registered (e.g. Campbell, or The Joanna Briggs Institute) together with any unique identification number assigned by them. If extracted data will be stored and made available through a repository such as the Systematic Review Data Repository (SRDR), details and a link should be included here. If none, leave blank.

34. Reference and/or URL for published protocol.

If the protocol for this review is published provide details (authors, title and journal details, preferably in Vancouver format)

1. Wan X, Wang W, Liu J, Tong T. Estimating the sample mean and standard deviation from the sample size, median, range and/or interquartile range. BMC Med Res Methodol. Dec 19 2014;14:135.
doi:10.1186/1471-2288-14-135

2. Tierney JF, Stewart LA, Ghersi D, Burdett S, Sydes MR. Practical methods for incorporating summary time-to-event data into meta-analysis. Trials. Jun 7 2007;8:16. doi:10.1186/1745-6215-8-16

3. Higgins JP, Altman DG, Gotzsche PC, et al. The Cochrane Collaboration's tool for assessing risk of bias in randomised trials. BMJ. Oct 18 2011;343:d5928. doi:10.1136/bmj.d5928

4. Wells GA SB, O'Connell D, Peterson J, Welch V, Losos M, Tugwell P. The Newcastle-Ottawa Scale (NOS) for assessing the quality of nonrandomized studies in meta-analysis. 2011;www.ohri.ca/programs/clinical_epidemiology/oxford.asp Accessed August 12, 2013

5. Sterne JA, Egger M, Smith GD. Systematic reviews in health care: Investigating and dealing with publication and other biases in meta-analysis. Bmj. Jul 14 2001;323(7304):101-5.

doi:10.1136/bmj.323.7304.101

Add web link to the published protocol.

Or, upload your published protocol here in pdf format. Note that the upload will be publicly accessible.

No I do not make this file publicly available until the review is complete

Please note that the information required in the PROSPERO registration form must be completed in full even if access to a protocol is given.

35. Dissemination plans.

Do you intend to publish the review on completion?

No

Give brief details of plans for communicating review findings.?

36. Keywords.

Give words or phrases that best describe the review. Separate keywords with a semicolon or new line. Keywords help PROSPERO users find your review (keywords do not appear in the public record but are included in searches). Be as specific and precise as possible. Avoid acronyms and abbreviations unless these are in wide use.

Tricuspid regurgitation, Tricuspid valve repair, Mitral valve surgery, Meta-analysis

37. Details of any existing review of the same topic by the same authors.

If you are registering an update of an existing review give details of the earlier versions and include a full bibliographic reference, if available.

38. * Current review status. [2 changes]

Update review status when the review is completed and when it is published.New registrations must be ongoing so this field is not editable for initial submission.

Please provide anticipated publication date

Review_Completed_not_published

39. Any additional information.

Provide any other information relevant to the registration of this review.

40. Details of final report/publication(s) or preprints if available.

Leave empty until publication details are available OR you have a link to a preprint (NOTE: this field is not editable for initial submission). List authors, title and journal details preferably in Vancouver format.

Give the link to the published review or preprint.
