# Supplementary material for: Tricuspid valve repair concomitant with mitral valve surgery: a systematic review and meta-analysis
Source: Int J Surg. 2023 Jun 7;109(7):2082–95. doi: 10.1097/JS9.0000000000000396 (PMC10389546; doi:10.1097/JS9.0000000000000396)

***Tricuspid valve repair concomitant with mitral valve surgery: a systematic review and meta-analysis***

**Supplementary Figures:**

**Subgroup analysis in Unmatched studies (S1-S5):**

S1 30-day mortality.

S2 Late mortality.

S3 Cardiac related mortality.

S4 Overall survival.

S5 Freedom from late TR.

**Secondary outcomes for the meta-analysis (S6-S12):**

S6 TR ⩽ moderate. S7 TR ⩾ moderate. S8 CPB time, min. S9 ACC time, min.

S10 Stroke. S11 PASP, mmHg. S12 LVEF, %.

**Subgroup analysis in RCT/Adjusted studies (S18-S27):**

S13 30-day mortality.

S14 Overall survival.

S15 Late mortality.

S16 Cardiac related mortality.

S17 Freedom from late TR.

# Subgroup analysis in Unmatched studies (S1-S5):

S1 30-day mortality.

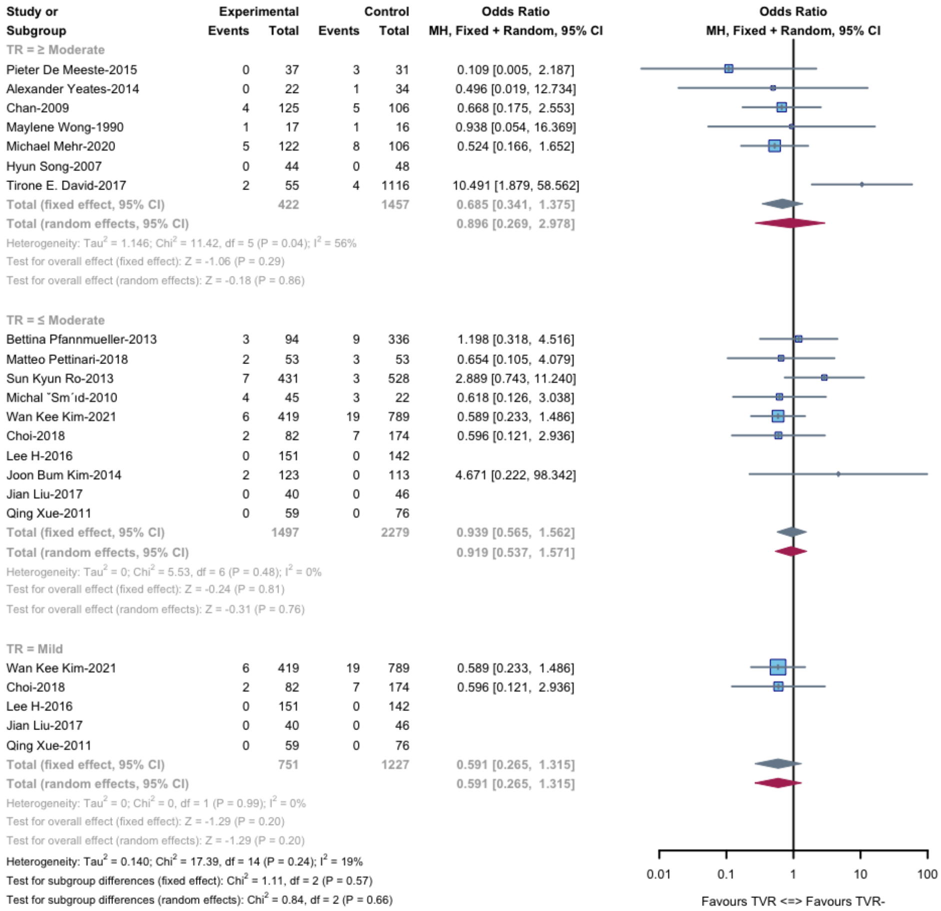

S2 Late mortality.

| Study or Subgroup | Experimental Events | Experimental Total | Control Events | Control Total | Odds Ratio MH, Fixed + Random, 95% CI |
|---|---|---|---|---|---|
| **TR = ≥ Moderate** | | | | | |
| Pieter De Meeste-2015 | 2 | 37 | 4 | 31 | 0.386 [0.066, 2.265] |
| Alexander Yeates-2014 | 6 | 22 | 5 | 34 | 2.175 [0.573, 8.261] |
| Michael Mehr-2020 | 20 | 122 | 33 | 106 | 0.434 [0.231, 0.816] |
| Hyun Song-2007 | 2 | 44 | 2 | 48 | 1.095 [0.148, 8.126] |
| **Total (fixed effect, 95% CI)** | | **225** | | **219** | **0.590 [0.353, 0.986]** |
| **Total (random effects, 95% CI)** | | | | | **0.716 [0.305, 1.682]** |
| Heterogeneity: $Tau^2$ = 0.318; $Chi^2$ = 5.17, df = 3 (P = 0.16); $I^2$ = 42% | | | | | |
| Test for overall effect (fixed effect): Z = -2.01 (P = 0.04) | | | | | |
| Test for overall effect (random effects): Z = -0.77 (P = 0.44) | | | | | |
| **TR = ≤ Moderate** | | | | | |
| Sun Kyun Ro-2013 | 64 | 431 | 38 | 528 | 2.249 [1.472, 3.435] |
| Michal ˇSm´ıd-2010 | 7 | 45 | 4 | 22 | 0.829 [0.215, 3.199] |
| Wan Kee Kim-2021 | 55 | 419 | 96 | 789 | 1.091 [0.765, 1.555] |
| HOU-1-2017 | 0 | 45 | 1 | 47 | 0.341 [0.014, 8.582] |
| HOU-2-2017 | 0 | 40 | 1 | 47 | 0.383 [0.015, 9.658] |
| Lee H-2016 | 5 | 151 | 10 | 142 | 0.452 [0.151, 1.357] |
| Joon Bum Kim-2014 | 5 | 123 | 9 | 113 | 0.490 [0.159, 1.508] |
| Lee H-2020 | 7 | 85 | 5 | 66 | 1.095 [0.331, 3.619] |
| Jian Liu-2017 | 0 | 40 | 2 | 46 | 0.220 [0.010, 4.715] |
| Qing Xue-2011 | 2 | 59 | 3 | 76 | 0.854 [0.138, 5.282] |
| **Total (fixed effect, 95% CI)** | | **1438** | | **1876** | **1.221 [0.964, 1.547]** |
| **Total (random effects, 95% CI)** | | | | | **0.969 [0.614, 1.529]** |
| Heterogeneity: $Tau^2$ = 0.181; $Chi^2$ = 16.84, df = 9 (P = 0.05); $I^2$ = 47% | | | | | |
| Test for overall effect (fixed effect): Z = 1.66 (P = 0.10) | | | | | |
| Test for overall effect (random effects): Z = -0.14 (P = 0.89) | | | | | |
| **TR = Mild** | | | | | |
| Wan Kee Kim-2021 | 55 | 419 | 96 | 789 | 1.091 [0.765, 1.555] |
| Lee H-2016 | 5 | 151 | 10 | 142 | 0.452 [0.151, 1.357] |
| Lee H-2020 | 7 | 85 | 5 | 66 | 1.095 [0.331, 3.619] |
| Jian Liu-2017 | 0 | 40 | 2 | 46 | 0.220 [0.010, 4.715] |
| Qing Xue-2011 | 2 | 59 | 3 | 76 | 0.854 [0.138, 5.282] |
| **Total (fixed effect, 95% CI)** | | **754** | | **1119** | **0.976 [0.711, 1.339]** |
| **Total (random effects, 95% CI)** | | | | | **0.989 [0.719, 1.359]** |
| Heterogeneity: $Tau^2$ = 0; $Chi^2$ = 3.23, df = 4 (P = 0.52); $I^2$ = 0% | | | | | |
| Test for overall effect (fixed effect): Z = -0.15 (P = 0.88) | | | | | |
| Test for overall effect (random effects): Z = -0.07 (P = 0.94) | | | | | |

Heterogeneity: $Tau^2$ = 0.143; $Chi^2$ = 31.77, df = 18 (P = 0.02); $I^2$ = 43%

Test for subgroup differences (fixed effect): $Chi^2$ = 6.61, df = 2 (P = 0.04)

Test for subgroup differences (random effects): $Chi^2$ = 0.49, df = 2 (P = 0.78)

**Odds Ratio**
**MH, Fixed + Random, 95% CI**

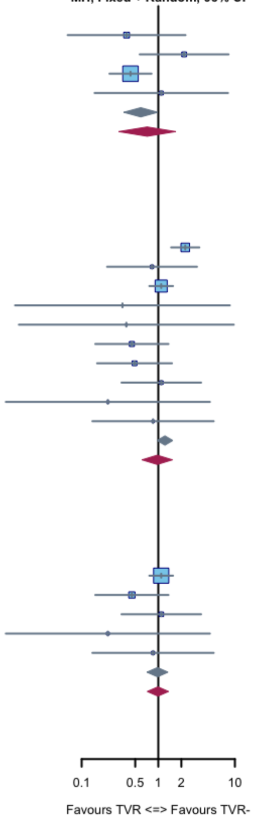

S3 Cardiac related mortality.

| Study or Subgroup | Experimental Events | Experimental Total | Control Events | Control Total | Odds Ratio MH, Fixed + Random, 95% CI |
|---|---|---|---|---|---|
| **TR = ≥ Moderate** | | | | | |
| Pieter De Meeste-2015 | 7 | 37 | 7 | 31 | 0.800 [0.247, 2.596] |
| Test for overall effect (fixed effect): Z = -0.37 (P = 0.71) | | | | | |
| Test for overall effect (random effects): Z = -0.37 (P = 0.71) | | | | | |
| **TR = ≤ Moderate** | | | | | |
| Matteo Pettinari-2018 | 3 | 53 | 5 | 53 | 0.576 [0.130, 2.543] |
| Lee H-2016 | 2 | 151 | 8 | 142 | 0.225 [0.047, 1.077] |
| Joon Bum Kim-2014 | 4 | 123 | 6 | 113 | 0.599 [0.165, 2.182] |
| Lee H-2020 | 2 | 85 | 3 | 66 | 0.506 [0.082, 3.120] |
| **Total (fixed effect, 95% CI)** | | **412** | | **374** | **0.443 [0.211, 0.929]** |
| **Total (random effects, 95% CI)** | | | | | **0.459 [0.216, 0.976]** |
| Heterogeneity: $Tau^2 = 0$; $Chi^2 = 1.07$, df = 3 (P = 0.78); $I^2 = 0\%$ | | | | | |
| Test for overall effect (fixed effect): Z = -2.15 (P = 0.03) | | | | | |
| Test for overall effect (random effects): Z = -2.02 (P = 0.04) | | | | | |
| **TR = Mild** | | | | | |
| Lee H-2016 | 2 | 151 | 8 | 142 | 0.225 [0.047, 1.077] |
| Lee H-2020 | 2 | 85 | 3 | 66 | 0.506 [0.082, 3.120] |
| **Total (fixed effect, 95% CI)** | | **236** | | **208** | **0.306 [0.095, 0.982]** |
| **Total (random effects, 95% CI)** | | | | | **0.318 [0.097, 1.041]** |
| Heterogeneity: $Tau^2 = 0$; $Chi^2 = 0.44$, df = 1 (P = 0.51); $I^2 = 0\%$ | | | | | |
| Test for overall effect (fixed effect): Z = -1.99 (P = 0.05) | | | | | |
| Test for overall effect (random effects): Z = -1.89 (P = 0.06) | | | | | |
| Heterogeneity: $Tau^2 = 0$; $Chi^2 = 2.72$, df = 6 (P = 0.84); $I^2 = 0\%$ | | | | | |
| Test for subgroup differences (fixed effect): $Chi^2 = 1.33$, df = 2 (P = 0.51) | | | | | |
| Test for subgroup differences (random effects): $Chi^2 = 1.20$, df = 2 (P = 0.55) | | | | | |

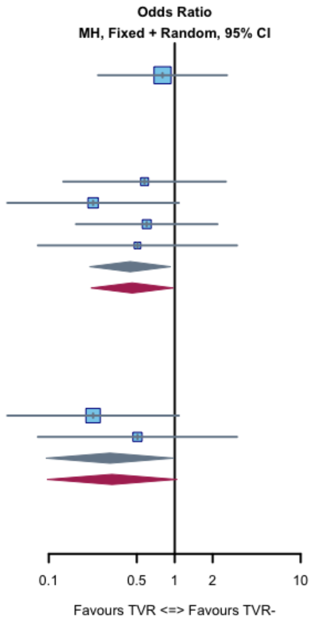

S4 Overall survival.

| Study or Subgroup | TE | SE | Hazard Ratio IV, Fixed + Random, 95% CI |
|---|---|---|---|
| **TR = ≥ Moderate** | | | |
| Pieter De Meeste-2015 | -1.079 | 0.6691 | 0.340 [0.092, 1.262] |
| Chan-2009 | 0.000 | 0.3406 | 1.000 [0.513, 1.949] |
| Michael Mehr-2020 | -0.598 | 0.2803 | 0.550 [0.318, 0.953] |
| Hyun Song-2007 | -0.446 | 0.5278 | 0.640 [0.227, 1.801] |
| Tirone E. David-2017 | -0.073 | 0.3863 | 0.930 [0.436, 1.983] |
| **Total (fixed effect, 95% CI)** | | | **0.699 [0.499, 0.980]** |
| **Total (random effects, 95% CI)** | | | **0.699 [0.499, 0.980]** |
| Heterogeneity: $Tau^2 = 0$; $Chi^2 = 3.57$, df = 4 (P = 0.47); $I^2 = 0\%$ | | | |
| Test for overall effect (fixed effect): Z = -2.08 (P = 0.04) | | | |
| Test for overall effect (random effects): Z = -2.08 (P = 0.04) | | | |
| **TR = ≤ Moderate** | | | |
| Bettina Pfannmueller-2013 | 0.859 | 0.3990 | 2.360 [1.080, 5.158] |
| Sun Kyun Ro-2013 | 0.565 | 0.2086 | 1.760 [1.169, 2.649] |
| Wan Kee Kim-2021 | -0.051 | 0.1692 | 0.950 [0.682, 1.324] |
| Choi-2018 | -0.400 | 0.7926 | 0.670 [0.142, 3.168] |
| Joon Bum Kim-2014 | -0.562 | 0.7118 | 0.570 [0.141, 2.300] |
| Lee H-2020 | -0.060 | 0.5418 | 0.942 [0.326, 2.724] |
| Qing Xue-2011 | -0.755 | 1.1248 | 0.470 [0.052, 4.261] |
| **Total (fixed effect, 95% CI)** | | | **1.216 [0.965, 1.532]** |
| **Total (random effects, 95% CI)** | | | **1.207 [0.815, 1.786]** |
| Heterogeneity: $Tau^2 = 0.100$; $Chi^2 = 10.67$, df = 6 (P = 0.10); $I^2 = 44\%$ | | | |
| Test for overall effect (fixed effect): Z = 1.66 (P = 0.10) | | | |
| Test for overall effect (random effects): Z = 0.94 (P = 0.35) | | | |
| **TR = Mild** | | | |
| Wan Kee Kim-2021 | -0.051 | 0.1692 | 0.950 [0.682, 1.324] |
| Choi-2018 | -0.400 | 0.7926 | 0.670 [0.142, 3.168] |
| Lee H-2020 | -0.060 | 0.5418 | 0.942 [0.326, 2.724] |
| Qing Xue-2011 | -0.755 | 1.1248 | 0.470 [0.052, 4.261] |
| **Total (fixed effect, 95% CI)** | | | **0.924 [0.679, 1.256]** |
| **Total (random effects, 95% CI)** | | | **0.924 [0.679, 1.256]** |
| Heterogeneity: $Tau^2 = 0$; $Chi^2 = 0.55$, df = 3 (P = 0.91); $I^2 = 0\%$ | | | |
| Test for overall effect (fixed effect): Z = -0.51 (P = 0.61) | | | |
| Test for overall effect (random effects): Z = -0.51 (P = 0.61) | | | |

Heterogeneity: $Tau^2 = 0.057$; $Chi^2 = 22.11$, df = 15 (P = 0.10); $I^2 = 32\%$
Test for subgroup differences (fixed effect): $Chi^2 = 7.32$, df = 2 (P = 0.03)
Test for subgroup differences (random effects): $Chi^2 = 4.32$, df = 2 (P = 0.12)

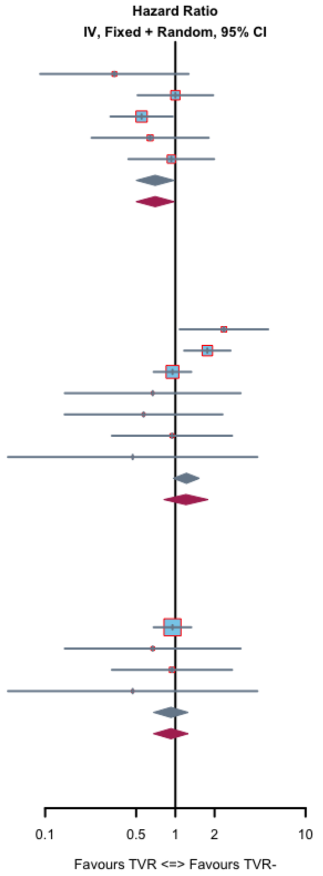

S5 Freedom from late TR.

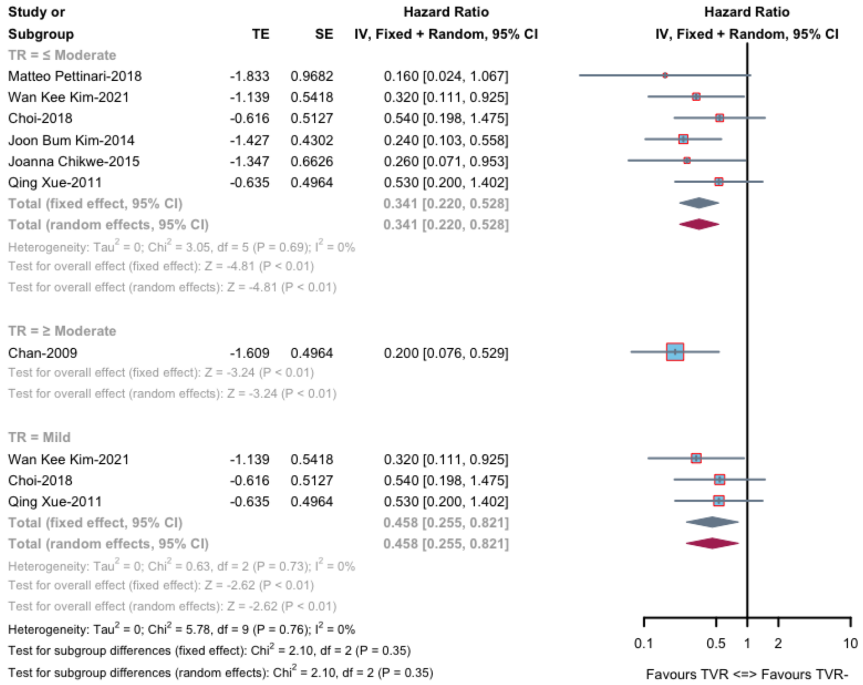

**Secondary outcomes for the meta-analysis (S6-S12):**

S6 TR ⩽ moderate.

| Study or Subgroup | Experimental Events | Experimental Total | Control Events | Control Total | Odds Ratio MH, Fixed + Random, 95% CI |
|---|---|---|---|---|---|
| Result = RCT/Adjusted study | | | | | |
| Umberto Benedetto-2012 | 21 | 22 | 15 | 22 | 9.800 [ 1.089, 88.229] |
| Philippe B. Bertrand-2014 | 44 | 45 | 29 | 33 | 6.069 [ 0.646, 57.059] |
| Kai-hu Shi-2012 | 41 | 70 | 25 | 97 | 4.072 [ 2.108, 7.863] |
| Chao Xv-2019 | 17 | 18 | 14 | 18 | 4.857 [ 0.486, 48.574] |
| Song-2016 | 50 | 50 | 50 | 50 | |
| Yundan Deng-2016 | 18 | 18 | 15 | 18 | 8.355 [ 0.400, 174.498] |
| Yongbo Li-2015 | 17 | 18 | 15 | 18 | 3.400 [ 0.319, 36.272] |
| Zadok-sig TR-2019 | 86 | 109 | 21 | 43 | 3.917 [ 1.842, 8.330] |
| Zadok- non-sig TR-2019 | 27 | 29 | 76 | 104 | 4.974 [ 1.110, 22.296] |
| Total (fixed effect, 95% CI) | | 379 | | 403 | 4.470 [ 2.904, 6.880] |
| Total (random effects, 95% CI) | | | | | 4.354 [ 2.830, 6.698] |
| Heterogeneity: $Tau^2 = 0$; $Chi^2 = 1$, df = 7 (P = 0.99); $I^2 = 0\%$ | | | | | |
| Test for overall effect (fixed effect): Z = 6.80 (P < 0.01) | | | | | |
| Test for overall effect (random effects): Z = 6.69 (P < 0.01) | | | | | |
| Result = Unmatched study | | | | | |
| Yue Zhong-2021 | 123 | 124 | 45 | 46 | 2.733 [ 0.167, 44.624] |
| Zadok-2019 | 102 | 138 | 106 | 147 | 1.096 [ 0.649, 1.850] |
| Jigang He-1-2012 | 25 | 27 | 49 | 50 | 0.255 [ 0.022, 2.951] |
| Jigang He-2-2012 | 12 | 12 | 49 | 50 | 0.758 [ 0.029, 19.738] |
| Gilles D. Dreyfus-2021 | 234 | 234 | 207 | 207 | |
| Jigang He-2014 | 38 | 40 | 49 | 50 | 0.388 [ 0.034, 4.438] |
| Bo Xiang-2009 | 50 | 56 | 51 | 56 | 0.817 [ 0.234, 2.850] |
| Michael Mehr-2020 | 99 | 122 | 0 | 106 | 901.851 [54.056, 15046.301] |
| Hyun Song-2007 | 43 | 44 | 22 | 48 | 50.818 [ 6.462, 399.643] |
| Jian Liu-2017 | 37 | 40 | 37 | 46 | 3.000 [ 0.752, 11.970] |
| Qing Xue-2011 | 57 | 59 | 71 | 76 | 2.007 [ 0.375, 10.731] |
| Gilles D. Dreyfus-2005 | 147 | 148 | 108 | 163 | 74.861 [10.200, 549.426] |
| Total (fixed effect, 95% CI) | | 1044 | | 1045 | 4.923 [ 3.653, 6.635] |
| Total (random effects, 95% CI) | | | | | 3.762 [ 0.945, 14.983] |
| Heterogeneity: $Tau^2 = 4.320$; $Chi^2 = 77.62$, df = 10 (P < 0.01); $I^2 = 87\%$ | | | | | |
| Test for overall effect (fixed effect): Z = 10.47 (P < 0.01) | | | | | |
| Test for overall effect (random effects): Z = 1.88 (P = 0.06) | | | | | |
| Heterogeneity: $Tau^2 = 1.583$; $Chi^2 = 77.31$, df = 18 (P < 0.01); $I^2 = 77\%$ | | | | | |
| Test for subgroup differences (fixed effect): $Chi^2 = 0.13$, df = 1 (P = 0.72) | | | | | |
| Test for subgroup differences (random effects): $Chi^2 = 0.04$, df = 1 (P = 0.84) | | | | | |

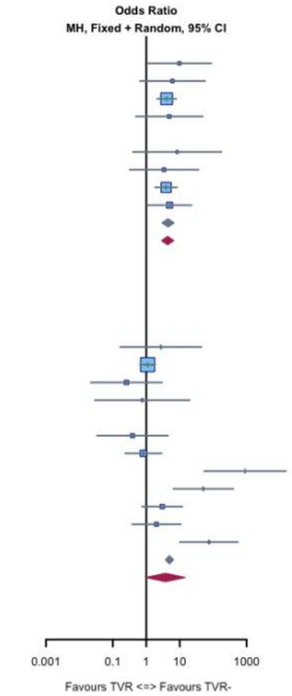


S7 TR ⩾ moderate.

| Study or Subgroup | Experimental Events | Experimental Total | Control Events | Control Total | Odds Ratio MH, Fixed + Random, 95% CI |
|---|---|---|---|---|---|
| Result = RCT/Adjusted study | | | | | |
| Umberto Benedetto-2012 | 0 | 22 | 6 | 22 | 0.056 [0.003, 1.073] |
| Philippe B. Bertrand-2014 | 1 | 45 | 4 | 33 | 0.165 [0.018, 1.549] |
| Kai-hu Shi-2012 | 13 | 70 | 24 | 97 | 0.694 [0.325, 1.481] |
| Chao Xv-2019 | 1 | 18 | 4 | 18 | 0.206 [0.021, 2.059] |
| Song-2016 | 0 | 50 | 0 | 50 | |
| Yundan Deng-2016 | 0 | 18 | 2 | 18 | 0.178 [0.008, 3.992] |
| Yongbo Li-2015 | 1 | 18 | 3 | 18 | 0.294 [0.028, 3.138] |
| Zadok-sig TR-2019 | 7 | 109 | 7 | 43 | 0.353 [0.116, 1.076] |
| Zadok- non-sig TR-2019 | 0 | 29 | 7 | 104 | 0.220 [0.012, 3.973] |
| Total (fixed effect, 95% CI) | | 379 | | 403 | 0.379 [0.225, 0.639] |
| Total (random effects, 95% CI) | | | | | 0.414 [0.242, 0.709] |
| Heterogeneity: $Tau^2 = 0$; $Chi^2 = 5.27$, df = 7 (P = 0.63); $I^2 = 0\%$ | | | | | |
| Test for overall effect (fixed effect): Z = -3.64 (P < 0.01) | | | | | |
| Test for overall effect (random effects): Z = -3.21 (P < 0.01) | | | | | |
| Result = Unmatched study | | | | | |
| Yue Zhong-2021 | 1 | 124 | 1 | 46 | 0.366 [0.022, 5.973] |
| Zadok-2019 | 7 | 138 | 14 | 147 | 0.508 [0.199, 1.298] |
| Jigang He-1-2012 | 2 | 27 | 1 | 50 | 3.920 [0.339, 45.350] |
| Jigang He-2-2012 | 0 | 12 | 1 | 50 | 1.320 [0.051, 34.392] |
| Gilles D. Dreyfus-2021 | 0 | 234 | 0 | 207 | |
| Jigang He-2014 | 2 | 40 | 1 | 50 | 2.579 [0.225, 29.516] |
| Bo Xiang-2009 | 6 | 56 | 5 | 56 | 1.224 [0.351, 4.269] |
| Michael Mehr-2020 | 25 | 122 | 106 | 106 | 0.001 [0.000, 0.020] |
| Hyun Song-2007 | 1 | 44 | 26 | 48 | 0.020 [0.003, 0.155] |
| Jian Liu-2017 | 3 | 40 | 9 | 46 | 0.333 [0.084, 1.330] |
| Qing Xue-2011 | 2 | 59 | 5 | 76 | 0.498 [0.093, 2.664] |
| Gilles D. Dreyfus-2005 | 1 | 148 | 55 | 163 | 0.013 [0.002, 0.098] |
| Total (fixed effect, 95% CI) | | 1044 | | 1045 | 0.120 [0.082, 0.177] |
| Total (random effects, 95% CI) | | | | | 0.250 [0.064, 0.980] |
| Heterogeneity: $Tau^2 = 4.165$; $Chi^2 = 61.5$, df = 10 (P < 0.01); $I^2 = 84\%$ | | | | | |
| Test for overall effect (fixed effect): Z = -10.83 (P < 0.01) | | | | | |
| Test for overall effect (random effects): Z = -1.99 (P = 0.05) | | | | | |
| Heterogeneity: $Tau^2 = 2.016$; $Chi^2 = 67.78$, df = 18 (P < 0.01); $I^2 = 73\%$ | | | | | |
| Test for subgroup differences (fixed effect): $Chi^2 = 12.03$, df = 1 (P < 0.01) | | | | | |
| Test for subgroup differences (random effects): $Chi^2 = 0.45$, df = 1 (P = 0.50) | | | | | |

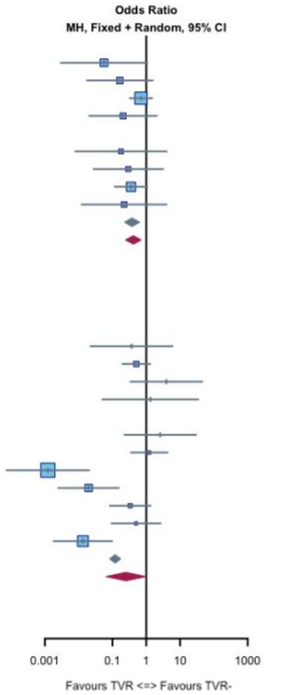

# S8 CPB time, min.

| Study or Subgroup | Experimental Mean | Experimental SD | Experimental Total | Control Mean | Control SD | Control Total | Mean Difference IV, Fixed + Random, 95% CI |
|---|---|---|---|---|---|---|---|
| Result = RCT/Adjusted study | | | | | | | |
| Antonio M. Calafiore-2009 | 111.000 | 32.0000 | 51 | 101.000 | 40.0000 | 59 | 10.000 [ -3.465, 23.465] |
| Ling-Chen Huang-2021 | 169.480 | 25.9600 | 40 | 153.100 | 23.0000 | 40 | 16.380 [ 5.632, 27.128] |
| Umberto Benedetto-2012 | 139.000 | 37.0000 | 22 | 120.000 | 66.0000 | 22 | 19.000 [-12.617, 50.617] |
| Philippe B. Bertrand-2014 | 219.000 | 68.0000 | 45 | 210.000 | 82.0000 | 33 | 9.000 [-25.314, 43.314] |
| Arman Kilic-2019 | 171.000 | 47.0000 | 119 | 137.000 | 40.0000 | 119 | 34.000 [ 22.911, 45.089] |
| Chao Xv-2019 | 203.000 | 56.0000 | 18 | 189.000 | 33.0000 | 18 | 14.000 [-16.028, 44.028] |
| Choi-PS matched-2018 | 238.000 | 57.0000 | 72 | 197.000 | 61.0000 | 72 | 41.000 [ 21.716, 60.284] |
| Song-2016 | 105.410 | 25.2400 | 50 | 99.520 | 26.8200 | 50 | 5.890 [ -4.318, 16.098] |
| Yundan Deng-2016 | 108.560 | 28.1000 | 18 | 98.330 | 25.7000 | 18 | 10.230 [ -7.362, 27.822] |
| Yongbo Li-2015 | 108.560 | 28.1000 | 18 | 98.330 | 25.7000 | 18 | 10.230 [ -7.362, 27.822] |
| Gammie-2021 | 166.100 | 69.3000 | 198 | 132.600 | 58.8000 | 203 | 33.500 [ 20.906, 46.094] |
| Total (fixed effect, 95% CI) | | | 651 | | | 652 | 19.074 [ 14.616, 23.532] |
| Total (random effects, 95% CI) | | | | | | | 19.105 [ 11.053, 27.157] |

Heterogeneity: $Tau^2$ = 105.765; $Chi^2$ = 27.74, df = 10 (P < 0.01); $I^2$ = 64%
Test for overall effect (fixed effect): Z = 8.39 (P < 0.01)
Test for overall effect (random effects): Z = 4.65 (P < 0.01)

| Study or Subgroup | Experimental Mean | Experimental SD | Experimental Total | Control Mean | Control SD | Control Total | Mean Difference IV, Fixed + Random, 95% CI |
|---|---|---|---|---|---|---|---|
| Result = Unmatched study | | | | | | | |
| Bettina Pfannmueller-2013 | 174.000 | 41.0000 | 94 | 124.000 | 43.0000 | 336 | 50.000 [ 40.522, 59.478] |
| Matteo Pettinari-2018 | 123.000 | 35.0000 | 53 | . | . | 53 | |
| Sun Kyun Ro-2013 | 146.300 | 55.9000 | 431 | 115.000 | 45.8000 | 528 | 31.300 [ 24.734, 37.866] |
| Gille Koppers-2012 | 202.000 | 58.0000 | 89 | 207.000 | 76.0000 | 86 | -5.000 [-25.080, 15.080] |
| Jerome Jouan-2015 | 128.000 | 50.0000 | 88 | 105.000 | 47.0000 | 113 | 23.000 [ 9.427, 36.573] |
| Chan-2009 | 140.000 | 43.0000 | 125 | 118.000 | 41.0000 | 106 | 22.000 [ 11.149, 32.851] |
| Choi-2018 | 237.000 | 55.0000 | 82 | 205.000 | 75.0000 | 174 | 32.000 [ 15.694, 48.306] |
| HOU-1-2017 | 108.600 | 12.4000 | 45 | 114.500 | 10.2000 | 47 | -5.900 [-10.551, -1.249] |
| HOU-2-2017 | 123.700 | 13.3000 | 40 | 114.500 | 10.2000 | 47 | 9.200 [ 4.151, 14.249] |
| Lee H-2016 | 116.000 | 25.0000 | 151 | 103.000 | 31.0000 | 142 | 13.000 [ 6.527, 19.473] |
| Joon Bum Kim-2014 | 131.860 | 45.2000 | 123 | 103.460 | 39.7000 | 113 | 28.400 [ 17.565, 39.235] |
| Gilles D. Dreyfus-2021 | 138.000 | 36.0000 | 234 | 125.000 | 50.0000 | 207 | 13.000 [ 4.774, 21.226] |
| Constance Verdonk-2017 | 94.000 | 32.0000 | 165 | 89.000 | 36.0000 | 122 | 5.000 [ -3.040, 13.040] |
| Total (fixed effect, 95% CI) | | | 1720 | | | 2074 | 12.894 [ 10.672, 15.115] |
| Total (random effects, 95% CI) | | | | | | | 18.018 [ 8.587, 27.449] |

Heterogeneity: $Tau^2$ = 249.088; $Chi^2$ = 178.58, df = 11 (P < 0.01); $I^2$ = 94%
Test for overall effect (fixed effect): Z = 11.37 (P < 0.01)
Test for overall effect (random effects): Z = 3.74 (P < 0.01)

Heterogeneity: $Tau^2$ = 215.700; $Chi^2$ = 212.24, df = 22 (P < 0.01); $I^2$ = 90%
Test for subgroup differences (fixed effect): $Chi^2$ = 5.91, df = 1 (P = 0.02)
Test for subgroup differences (random effects): $Chi^2$ = 0.03, df = 1 (P = 0.86)

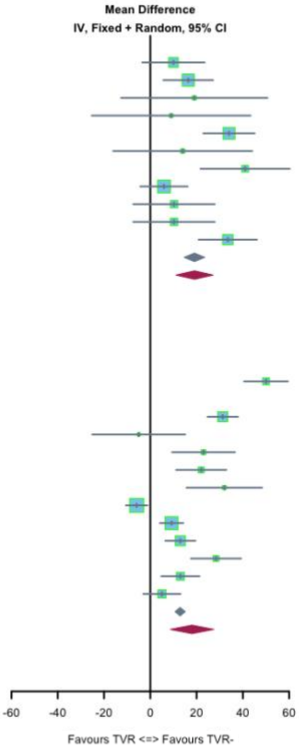


# S9 ACC time, min.

| Study or Subgroup | Experimental Mean | Experimental SD | Experimental Total | Control Mean | Control SD | Control Total | Mean Difference IV, Fixed + Random, 95% CI |
|---|---|---|---|---|---|---|---|
| Result = RCT/Adjusted study | | | | | | | |
| Antonio M. Calafiore-2009 | 89.000 | 36.0000 | 51 | 83.000 | 42.0000 | 59 | 6.000 [ -8.576, 20.576] |
| Ling-Chen Huang-2021 | 110.800 | 17.3700 | 40 | 101.000 | 14.3800 | 40 | 9.800 [ 2.812, 16.788] |
| Philippe B. Bertrand-2014 | 164.000 | 43.0000 | 45 | 151.000 | 66.0000 | 33 | 13.000 [-12.786, 38.786] |
| Arman Kilic-2019 | 125.000 | 37.0000 | 119 | 100.000 | 32.0000 | 119 | 25.000 [ 16.211, 33.789] |
| Chao Xv-2019 | 72.000 | 36.0000 | 18 | 64.000 | 32.0000 | 18 | 8.000 [-14.251, 30.251] |
| Choi-PS matched-2018 | 163.000 | 43.0000 | 72 | 136.000 | 45.0000 | 72 | 27.000 [ 12.623, 41.377] |
| Song-2016 | 70.870 | 21.7100 | 50 | 66.210 | 24.2800 | 50 | 4.660 [ -4.368, 13.688] |
| Yundan Deng-2016 | 71.940 | 26.4200 | 18 | 65.170 | 21.3400 | 18 | 6.770 [ -8.919, 22.459] |
| Yongbo Li-2015 | 71.940 | 26.4200 | 18 | 65.170 | 21.3400 | 18 | 6.770 [ -8.919, 22.459] |
| Total (fixed effect, 95% CI) | | | 431 | | | 427 | 12.425 [ 8.559, 16.292] |
| Total (random effects, 95% CI) | | | | | | | 12.300 [ 6.056, 18.543] |

Heterogeneity: $Tau^2$ = 43.365; $Chi^2$ = 17.09, df = 8 (P = 0.03); $I^2$ = 53%
Test for overall effect (fixed effect): Z = 6.30 (P < 0.01)
Test for overall effect (random effects): Z = 3.86 (P < 0.01)

| Study or Subgroup | Experimental Mean | Experimental SD | Experimental Total | Control Mean | Control SD | Control Total | Mean Difference IV, Fixed + Random, 95% CI |
|---|---|---|---|---|---|---|---|
| Result = Unmatched study | | | | | | | |
| Bettina Pfannmueller-2013 | 100.000 | 29.0000 | 94 | 71.000 | 35.0000 | 336 | 29.000 [ 22.045, 35.955] |
| Matteo Pettinari-2018 | 100.000 | 36.0000 | 53 | . | . | 53 | |
| Sun Kyun Ro-2013 | 95.900 | 35.9000 | 431 | 73.900 | 33.6000 | 528 | 22.000 [ 17.561, 26.439] |
| Gille Koppers-2012 | 152.000 | 49.0000 | 89 | 151.000 | 57.0000 | 86 | 1.000 [-14.772, 16.772] |
| Jerome Jouan-2015 | 92.000 | 36.0000 | 88 | 78.000 | 35.0000 | 113 | 14.000 [ 4.089, 23.911] |
| Chan-2009 | 85.000 | 20.0000 | 125 | 75.000 | 23.0000 | 106 | 10.000 [ 4.391, 15.609] |
| Choi-2018 | 162.000 | 42.0000 | 82 | 142.000 | 54.0000 | 174 | 20.000 [ 7.875, 32.125] |
| HOU-1-2017 | 64.500 | 8.3000 | 45 | 62.300 | 8.2000 | 47 | 2.200 [ -1.173, 5.573] |
| HOU-2-2017 | 65.100 | 8.7000 | 40 | 62.300 | 8.2000 | 47 | 2.800 [ -0.773, 6.373] |
| Lee H-2016 | 97.000 | 24.0000 | 151 | 77.000 | 30.0000 | 142 | 20.000 [ 13.755, 26.245] |
| Joon Bum Kim-2014 | 87.960 | 30.5000 | 123 | 64.660 | 30.9000 | 113 | 23.300 [ 15.457, 31.143] |
| Gilles D. Dreyfus-2021 | 113.000 | 30.0000 | 234 | 99.000 | 38.0000 | 207 | 14.000 [ 7.552, 20.448] |
| Constance Verdonk-2017 | 72.000 | 28.0000 | 165 | 70.000 | 33.0000 | 122 | 2.000 [ -5.249, 9.249] |
| Total (fixed effect, 95% CI) | | | 1720 | | | 2074 | 10.643 [ 9.011, 12.275] |
| Total (random effects, 95% CI) | | | | | | | 13.449 [ 7.609, 19.290] |

Heterogeneity: $Tau^2$ = 91.104; $Chi^2$ = 123.84, df = 11 (P < 0.01); $I^2$ = 91%
Test for overall effect (fixed effect): Z = 12.78 (P < 0.01)
Test for overall effect (random effects): Z = 4.51 (P < 0.01)

Heterogeneity: $Tau^2$ = 80.061; $Chi^2$ = 141.62, df = 20 (P < 0.01); $I^2$ = 86%
Test for subgroup differences (fixed effect): $Chi^2$ = 0.69, df = 1 (P = 0.41)
Test for subgroup differences (random effects): $Chi^2$ = 0.07, df = 1 (P = 0.79)

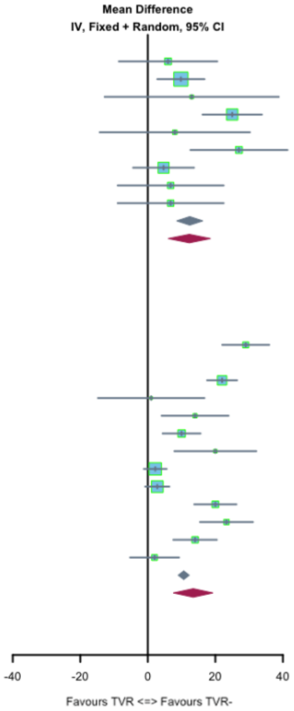

S10 Stroke.

| Study or Subgroup | Experimental Events | Experimental Total | Control Events | Control Total | Odds Ratio MH, Fixed + Random, 95% CI |
|---|---|---|---|---|---|
| Result = RCT/Adjusted study | | | | | |
| Ling-Chen Huang-2021 | 0 | 40 | 0 | 40 | |
| Arman Kilic-2019 | 2 | 119 | 2 | 119 | 1.000 [0.139, 7.218] |
| Choi-PS matched-2018 | 1 | 72 | 1 | 72 | 1.000 [0.061, 16.302] |
| Total (fixed effect, 95% CI) | | 231 | | 231 | 1.000 [0.199, 5.018] |
| Total (random effects, 95% CI) | | | | | 1.000 [0.199, 5.018] |
| Heterogeneity: $Tau^2 = 0$; $Chi^2 = 0$, df = 1 (P = 1.00); $I^2 = 0\%$ | | | | | |
| Test for overall effect (fixed effect): Z = 0.00 (P = 1.00) | | | | | |
| Test for overall effect (random effects): Z = 0.00 (P = 1.00) | | | | | |
| Result = Unmatched study | | | | | |
| Sun Kyun Ro-2013 | 13 | 431 | 17 | 528 | 0.935 [0.449, 1.947] |
| Choi-2018 | 2 | 82 | 1 | 174 | 4.325 [0.386, 48.398] |
| Lee H-2016 | 21 | 151 | 27 | 142 | 0.688 [0.369, 1.283] |
| Michael Mehr-2020 | 2 | 122 | 1 | 106 | 1.750 [0.156, 19.576] |
| Tirone E. David-2017 | 0 | 55 | 7 | 1116 | 1.333 [0.075, 23.632] |
| Gilles D. Dreyfus-2005 | 1 | 148 | 1 | 163 | 1.102 [0.068, 17.778] |
| Total (fixed effect, 95% CI) | | 989 | | 2229 | 0.876 [0.562, 1.363] |
| Total (random effects, 95% CI) | | | | | 0.871 [0.558, 1.361] |
| Heterogeneity: $Tau^2 = 0$; $Chi^2 = 2.71$, df = 5 (P = 0.74); $I^2 = 0\%$ | | | | | |
| Test for overall effect (fixed effect): Z = -0.59 (P = 0.56) | | | | | |
| Test for overall effect (random effects): Z = -0.61 (P = 0.54) | | | | | |

Heterogeneity: $Tau^2 = 0$; $Chi^2 = 2.74$, df = 7 (P = 0.91); $I^2 = 0\%$

Test for subgroup differences (fixed effect): $Chi^2 = 0.02$, df = 1 (P = 0.88)

Test for subgroup differences (random effects): $Chi^2 = 0.03$, df = 1 (P = 0.87)

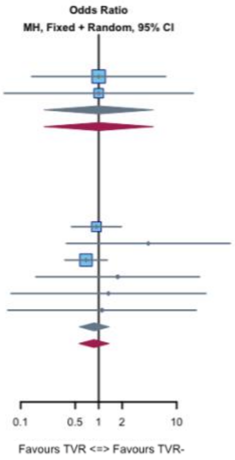


S11 PASP, mmHg.

| Study or Subgroup | Experimental Mean | Experimental SD | Experimental Total | Control Mean | Control SD | Control Total | Mean Difference IV, Fixed + Random, 95% CI |
|---|---|---|---|---|---|---|---|
| Result = RCT/Adjusted study | | | | | | | |
| Ling-Chen Huang-2021 | 33.650 | 7.3400 | 40 | 38.000 | 8.4500 | 40 | -4.350 [ -7.819, -0.881] |
| Umberto Benedetto-2012 | 41.000 | 8.0000 | 22 | 40.000 | 5.0000 | 22 | 1.000 [ -2.942, 4.942] |
| Philippe B. Bertrand-2014 | 37.000 | 9.0000 | 45 | 36.000 | 12.0000 | 33 | 1.000 [ -3.866, 5.866] |
| Zadok-sig TR-2019 | 45.000 | 13.0000 | 109 | 47.000 | 18.0000 | 43 | -2.000 [ -7.908, 3.908] |
| Zadok- non-sig TR-2019 | 44.000 | 13.0000 | 29 | 37.000 | 11.0000 | 104 | 7.000 [ 1.818, 12.182] |
| Total (fixed effect, 95% CI) | | | 245 | | | 242 | -0.202 [ -2.180, 1.776] |
| Total (random effects, 95% CI) | | | | | | | 0.376 [ -3.413, 4.166] |
| Heterogeneity: $Tau^2 = 13.047$; $Chi^2 = 13.86$, df = 4 (P < 0.01); $I^2 = 71\%$ | | | | | | | |
| Test for overall effect (fixed effect): Z = -0.20 (P = 0.84) | | | | | | | |
| Test for overall effect (random effects): Z = 0.19 (P = 0.85) | | | | | | | |
| Result = Unmatched study | | | | | | | |
| Matteo Pettinari-2018 | 22.960 | 14.3800 | 53 | 29.490 | 12.3300 | 53 | -6.530 [-11.630, -1.430] |
| Yue Zhong-2021 | 25.000 | 6.8000 | 124 | 24.700 | 5.3000 | 46 | 0.300 [ -1.644, 2.244] |
| Choi-2018 | 35.300 | 6.5000 | 82 | 34.300 | 7.0000 | 174 | 1.000 [ -0.750, 2.750] |
| HOU-1-2017 | 45.300 | 7.9000 | 45 | 47.600 | 8.3000 | 47 | -2.300 [ -5.610, 1.010] |
| HOU-2-2017 | 45.300 | 7.8000 | 40 | 47.600 | 8.3000 | 47 | -2.300 [ -5.687, 1.087] |
| Zadok-2019 | 45.000 | 13.0000 | 138 | 38.000 | 11.0000 | 147 | 7.000 [ 4.195, 9.805] |
| Total (fixed effect, 95% CI) | | | 482 | | | 514 | 0.678 [ -0.356, 1.712] |
| Total (random effects, 95% CI) | | | | | | | -0.104 [ -2.982, 2.774] |
| Heterogeneity: $Tau^2 = 10.459$; $Chi^2 = 33.55$, df = 5 (P < 0.01); $I^2 = 85\%$ | | | | | | | |
| Test for overall effect (fixed effect): Z = 1.29 (P = 0.20) | | | | | | | |
| Test for overall effect (random effects): Z = -0.07 (P = 0.94) | | | | | | | |

Heterogeneity: $Tau^2 = 9.876$; $Chi^2 = 48.00$, df = 10 (P < 0.01); $I^2 = 79\%$

Test for subgroup differences (fixed effect): $Chi^2 = 0.60$, df = 1 (P = 0.44)

Test for subgroup differences (random effects): $Chi^2 = 0.04$, df = 1 (P = 0.84)

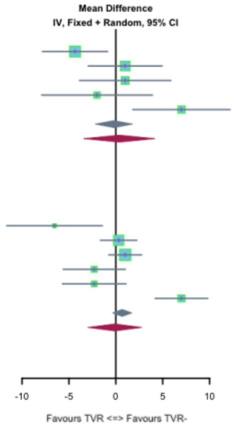


S12 LVEF, %.

| Study or Subgroup | Experimental Mean | Experimental SD | Experimental Total | Control Mean | Control SD | Control Total | Mean Difference IV, Fixed + Random, 95% CI |
|---|---|---|---|---|---|---|---|
| Result = RCT/Adjusted study | | | | | | | |
| Ling-Chen Huang-2021 | 57.830 | 7.0800 | 40 | 58.480 | 5.8700 | 40 | -0.650 [ -3.500, 2.200] |
| Umberto Benedetto-2012 | 50.000 | 8.0000 | 22 | 51.000 | 6.0000 | 22 | -1.000 [ -5.179, 3.179] |
| Philippe B. Bertrand-2014 | 50.000 | 16.0000 | 45 | 54.000 | 15.0000 | 33 | -4.000 [-10.931, 2.931] |
| Chao Xv-2019 | 59.700 | 10.8000 | 18 | 58.900 | 13.7000 | 18 | 0.800 [ -7.259, 8.859] |
| Yongbo Li-2015 | 59.060 | 13.1400 | 18 | 60.060 | 9.4500 | 18 | -1.000 [ -8.477, 6.477] |
| Total (fixed effect, 95% CI) | | | 143 | | | 131 | -0.964 [ -3.030, 1.101] |
| Total (random effects, 95% CI) | | | | | | | -0.964 [ -3.030, 1.101] |
| Heterogeneity: $Tau^2 = 0$; $Chi^2 = 0.97$, df = 4 (P = 0.91); $I^2 = 0\%$ | | | | | | | |
| Test for overall effect (fixed effect): Z = -0.92 (P = 0.36) | | | | | | | |
| Test for overall effect (random effects): Z = -0.92 (P = 0.36) | | | | | | | |
| Result = Unmatched study | | | | | | | |
| Matteo Pettinari-2018 | 47.000 | 9.0000 | 53 | 49.000 | 11.0000 | 53 | -2.000 [ -5.826, 1.826] |
| Michal ˇSm´ıd-2010 | 47.300 | 10.8000 | 45 | 41.300 | 14.9000 | 22 | 6.000 [ -0.980, 12.980] |
| Yue Zhong-2021 | 60.400 | 3.8000 | 124 | 60.400 | 3.3000 | 46 | 0.000 [ -1.165, 1.165] |
| HOU-1-2017 | 50.900 | 7.3000 | 45 | 48.700 | 8.1000 | 47 | 2.200 [ -0.948, 5.348] |
| HOU-2-2017 | 52.600 | 7.4000 | 40 | 48.700 | 8.1000 | 47 | 3.900 [ 0.641, 7.159] |
| Jigang He-1-2012 | 56.000 | 10.0000 | 27 | 59.000 | 9.0000 | 50 | -3.000 [ -7.522, 1.522] |
| Jigang He-2-2012 | 59.000 | 9.0000 | 12 | 59.000 | 9.0000 | 50 | 0.000 [ -5.670, 5.670] |
| Maylene Wong-1990 | 64.000 | 9.0000 | 17 | 64.000 | 10.0000 | 16 | 0.000 [ -6.505, 6.505] |
| Total (fixed effect, 95% CI) | | | 363 | | | 331 | 0.381 [ -0.562, 1.324] |
| Total (random effects, 95% CI) | | | | | | | 0.686 [ -1.017, 2.389] |
| Heterogeneity: $Tau^2 = 2.249$; $Chi^2 = 12.33$, df = 7 (P = 0.09); $I^2 = 43\%$ | | | | | | | |
| Test for overall effect (fixed effect): Z = 0.79 (P = 0.43) | | | | | | | |
| Test for overall effect (random effects): Z = 0.79 (P = 0.43) | | | | | | | |

Heterogeneity: $Tau^2 = 0.745$; $Chi^2 = 14.64$, df = 12 (P = 0.26); $I^2 = 18\%$

Test for subgroup differences (fixed effect): $Chi^2 = 1.35$, df = 1 (P = 0.25)

Test for subgroup differences (random effects): $Chi^2 = 1.46$, df = 1 (P = 0.23)

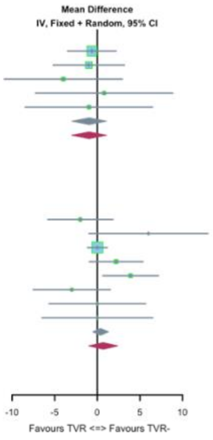

**Subgroup analysis in RCT/Adjusted studies (S13-S17):**

S13 30-day mortality.

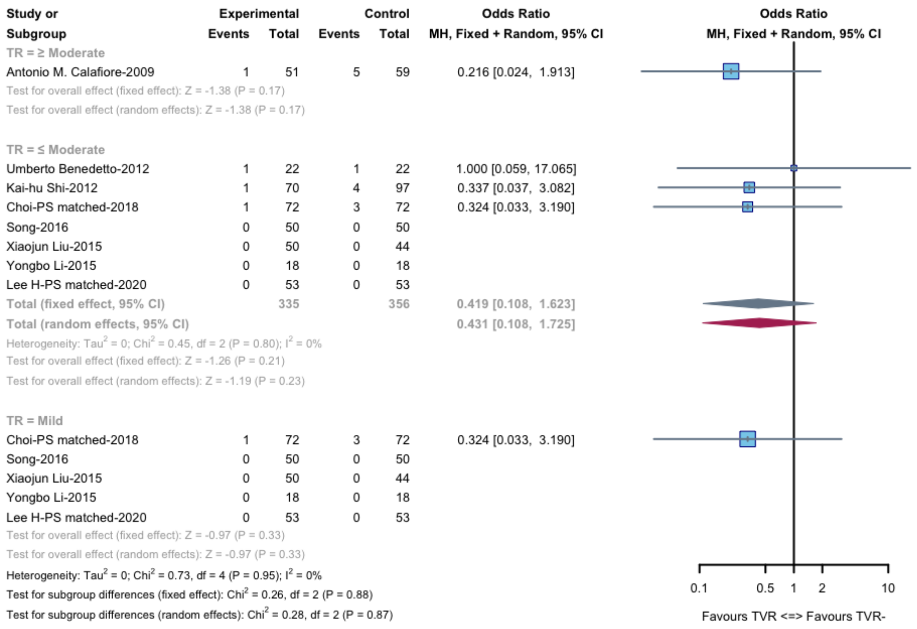


S14 Overall survival.

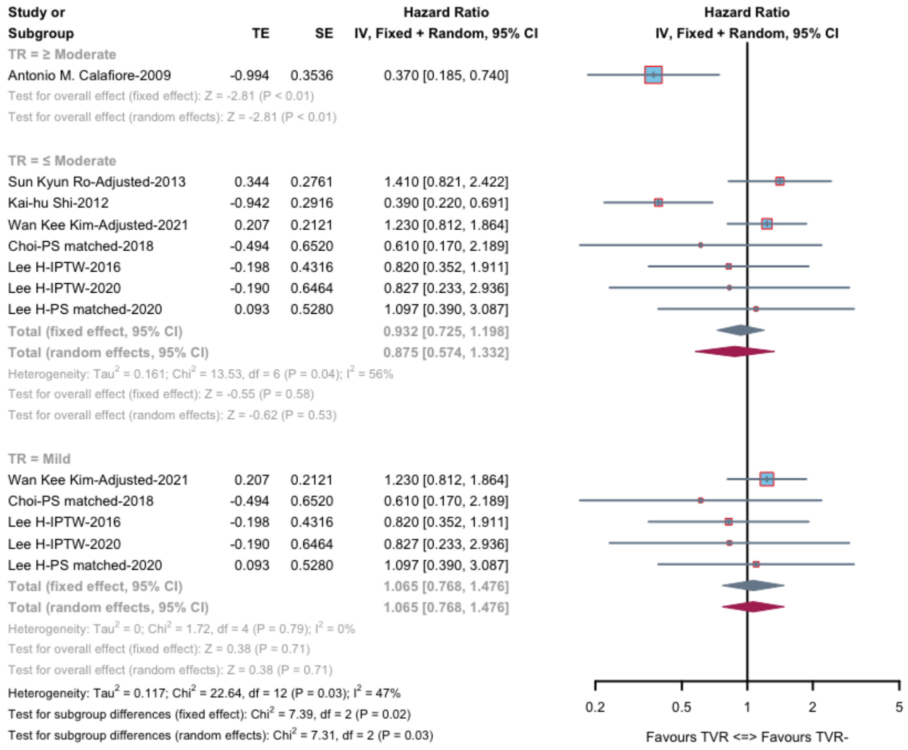

S15 Late mortality.

| Study | Experimental Events | Experimental Total | Control Events | Control Total | Weight (common) | Weight (random) | Odds Ratio MH, Fixed + Random, 95% CI |
|---|---|---|---|---|---|---|---|
| Kai-hu Shi-2012 | 16 | 70 | 44 | 97 | 63.9% | 57.3% | 0.357 [0.180, 0.709] |
| Song-2016 | 0 | 50 | 7 | 50 | 16.7% | 6.8% | 0.057 [0.003, 1.035] |
| Gammie-2021 | 6 | 198 | 9 | 203 | 19.4% | 35.9% | 0.674 [0.235, 1.929] |
| **Total (fixed effect, 95% CI)** | | **318** | | **350** | **100.0%** | **--** | **0.368 [0.212, 0.638]** |
| **Total (random effects, 95% CI)** | | | | | **--** | **100.0%** | **0.396 [0.181, 0.864]** |

Heterogeneity: $Tau^2 = 0.155$; $Chi^2 = 2.86$, df = 2 (P = 0.24); $I^2 = 30\%$
Test for overall effect (fixed effect): Z = -3.56 (P < 0.01)
Test for overall effect (random effects): Z = -2.33 (P = 0.02)

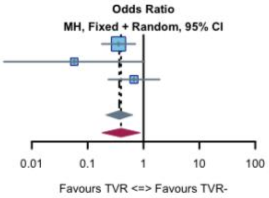


S16 Cardiac related mortality.

| Study | Experimental Events | Experimental Total | Control Events | Control Total | Weight (common) | Weight (random) | Odds Ratio MH, Fixed + Random, 95% CI |
|---|---|---|---|---|---|---|---|
| Antonio M. Calafiore-2009 | 13 | 51 | 30 | 59 | 44.2% | 44.5% | 0.331 [0.147, 0.744] |
| Umberto Benedetto-2012 | 1 | 22 | 0 | 22 | 1.0% | 2.8% | 3.140 [0.121, 81.354] |
| Kai-hu Shi-2012 | 12 | 70 | 37 | 97 | 54.8% | 52.8% | 0.336 [0.159, 0.706] |
| **Total (fixed effect, 95% CI)** | | **143** | | **178** | **100.0%** | **--** | **0.361 [0.212, 0.616]** |
| **Total (random effects, 95% CI)** | | | | | **--** | **100.0%** | **0.355 [0.206, 0.609]** |

Heterogeneity: $Tau^2 = 0$; $Chi^2 = 1.78$, df = 2 (P = 0.41); $I^2 = 0\%$
Test for overall effect (fixed effect): Z = -3.74 (P < 0.01)
Test for overall effect (random effects): Z = -3.76 (P < 0.01)

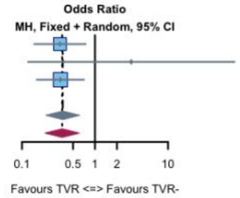


S17 Freedom from late TR.

| Study or Subgroup | TE | SE | Hazard Ratio IV, Fixed + Random, 95% CI |
|---|---|---|---|
| TR = Mild | | | |
| Wan Kee Kim-Adjusted-2021 | -0.446 | 0.5206 | 0.640 [0.231, 1.775] |
| Choi-PS matched-2018 | -2.207 | 0.9410 | 0.110 [0.017, 0.696] |
| Lee H-IPTW-2016 | -1.273 | 0.7004 | 0.280 [0.071, 1.105] |
| Lee H-PS matched-2020 | -1.207 | 0.6694 | 0.299 [0.081, 1.110] |
| **Total (fixed effect, 95% CI)** | | | **0.354 [0.185, 0.679]** |
| **Total (random effects, 95% CI)** | | | **0.354 [0.185, 0.679]** |

Heterogeneity: $Tau^2 = 0.002$; $Chi^2 = 3.01$, df = 3 (P = 0.39); $I^2 = 0\%$
Test for overall effect (fixed effect): Z = -3.13 (P < 0.01)
Test for overall effect (random effects): Z = -3.12 (P < 0.01)

Heterogeneity: $Tau^2 = 0.002$; $Chi^2 = 3.01$, df = 3 (P = 0.39); $I^2 = 0\%$
Test for subgroup differences (fixed effect): $Chi^2 = 0.00$, df = 0 (P = NA)
Test for subgroup differences (random effects): $Chi^2 = 0.00$, df = 0 (P = NA)

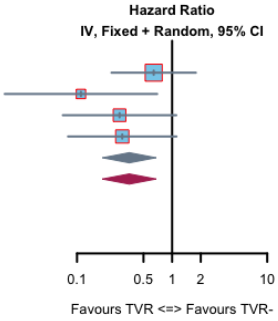

Supplement: Supplementary file 5 [file js9-109-2082-s005.pdf]
