# Supplementary figures and images for: Tricuspid valve repair concomitant with mitral valve surgery: a systematic review and meta-analysis
Source: Int J Surg. 2023 Jun 7;109(7):2082–95. doi: 10.1097/JS9.0000000000000396 (PMC10389546; doi:10.1097/JS9.0000000000000396)

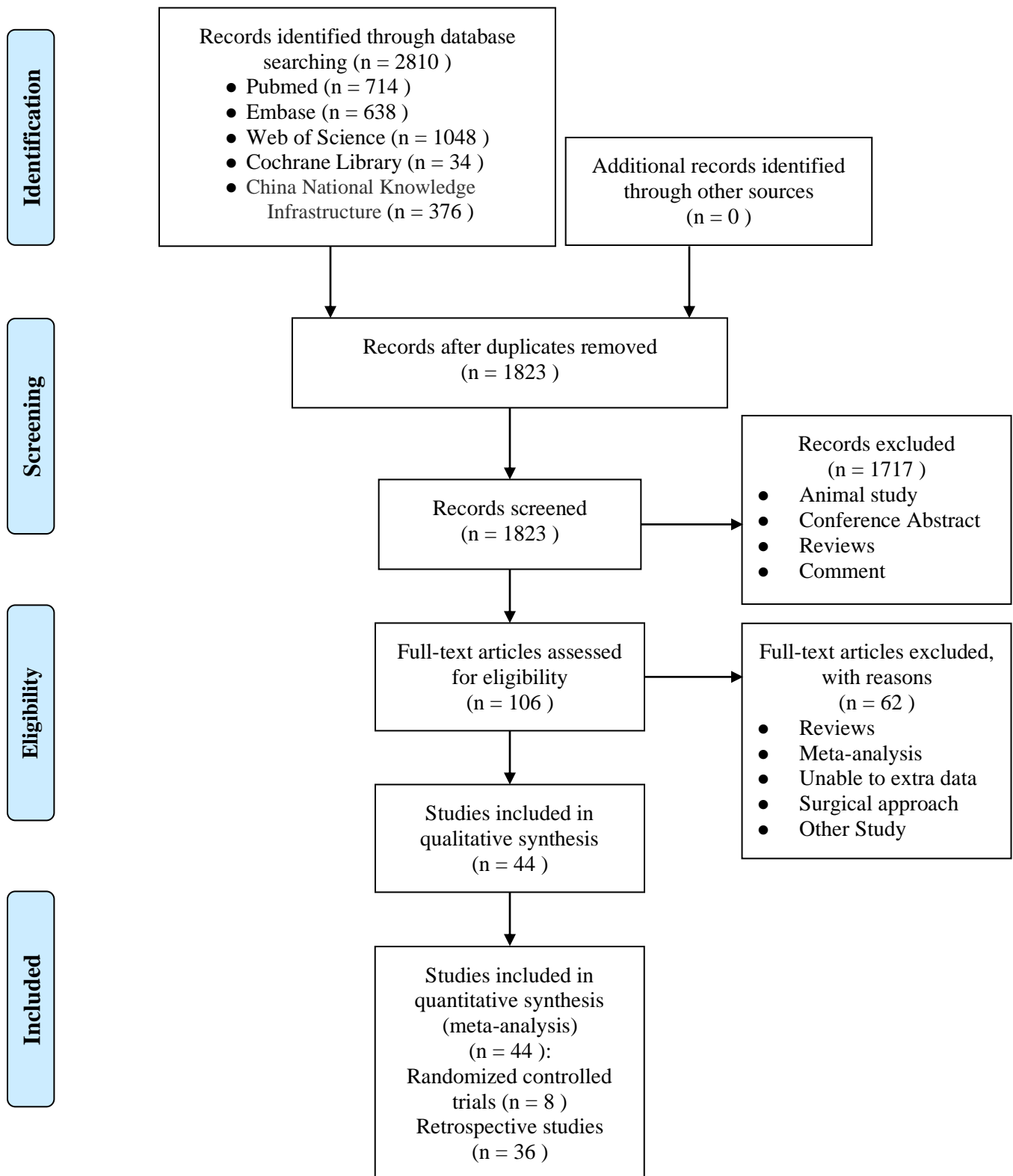

Supplement: Supplementary file 6 [file js9-109-2082-s006.pdf]
